# Integrated Psychosocial Care in Intensive Care (IPS-Pilot): Protocol for the Systematic, Multimethod Development of a Complex Intervention (Phase A)

**DOI:** 10.2196/65682

**Published:** 2025-06-06

**Authors:** Sophie Felicitas Nickel, Simone Korger, Wencke Schindler, Heike Heytens, Gironimo Krieg, Karl-Philipp Drewitz, Katrin Schürmann, Leon Schössow, Julianna Gehrig, Marius Binneböse, Christian Hirning, Klaus Hönig, Rolf Konstantin Niessen, Julia Kirschbaum, Laurence Erdur, Sophie Peter, Florian Junne, Matthias Rose, Christian Apfelbacher, Harald Gündel

**Affiliations:** 1 Clinic of Psychosomatic Medicine and Psychotherapy University Hospital Ulm Ulm Germany; 2 Institute of Social Medicine and Health Systems Research (ISMHSR) Medical Faculty Otto von Guericke University Magdeburg Magdeburg Germany; 3 Department of Psychosomatic Medicine Charité Berlin Germany; 4 Psychosomatic Medicine and Psychosomatic Therapy Medical Faculty University Hospital Magdeburg Magdeburg Germany

**Keywords:** health services research, psychosocial intervention, health personnel, intensive care units, critical care, complex intervention, feasibility study

## Abstract

**Background:**

There is a high, co-dependent strain on health care professionals (HCPs), patients, and their relatives in intensive care units (ICUs), leading to long-term mental, physical, and occupational consequences. To date, there is no systematic intervention to address this growing problem.

**Objective:**

The aim of the IPS-Pilot (German: Integrierte Psychosoziale Versorgung; English: “Integrated Psychosocial Care”) project is the development (phase A) and pilot testing (phase B) of an integrated and complex psychosocial care intervention for HCPs, patients, and their relatives in ICUs. This study protocol focuses on phase A. A separate protocol for phase B will be published later.

**Methods:**

A structured, multimethod approach was used to gather evidence from the target groups mentioned above on the needs, expected benefits, and necessary conditions for implementation. These methods included (1) a scoping umbrella review conducted by 2 researchers, who independently screened and selected reviews and meta-analyses in the field of needs and demands in the ICU setting, following the Levac framework; (2) web-based and face-to-face interviews and focus group discussions, which were coded independently by 2 researchers and analyzed using qualitative content analysis; the identified categories and codes were then quantified by (3) an online survey conducted with former ICU patients, their relatives, HCPs working in ICUs, and members of the general population. Synthesized results—complemented by the theories of Psychosocial Safety Climate and Conservation of Resources, as well as online and face-to-face stakeholder workshops—were used for intervention development, which was guided by the Intervention Mapping framework.

**Results:**

Through the 4 substudies, we aim to gain insights into the psychosocial needs of the aforementioned target groups. Intermediate analyses were conducted to develop both a model of the problem and a model illustrating how these needs can be effectively addressed through a psychosocial intervention that involves integrating a clinically trained psychologist into the HCP team. Data collection and analysis for the intervention development were completed by June 2024, including substudy 1 (n=104 included articles), substudy 2 (n=22 interviews and n=18 participants in focus groups), substudy 3 (n=237 survey participants), and substudy 4 (n=11, n=15, and n=20 participants in 3 consecutive workshops), with further analyses still ongoing beyond the scope of intervention development. The results of the substudies, as well as the final needs-based intervention design, will be published separately. These findings form the basis for the feasibility study (phase B), conducted from July 2024 to July 2025, during which the intervention will be implemented in randomly selected ICU wards and evaluated in terms of feasibility.

**Conclusions:**

Phase B will assess the feasibility of the IPS intervention. The findings will be incorporated into the intervention design and serve as the basis for a future randomized controlled trial to evaluate its efficacy.

**Trial Registration:**

OSF Registries 10.17605/OSF.IO/VFXJK; https://osf.io/vfxjk

**International Registered Report Identifier (IRRID):**

RR1-10.2196/65682

## Introduction

### Background

The German health care system is well equipped in terms of the number of intensive care unit (ICU) beds. Compared with other European countries, Germany had the highest average rate, with 29.9 ICU beds per 100,000 population during the COVID-19 pandemic [1]. However, on a global scale, there is a considerable shortage of ICU-qualified nurses and doctors. In accordance with the legal requirements for minimum care staffing in care-sensitive settings in Germany (Pflegepersonaluntergrenzenverordnung), and the recommendations of the German Interdisciplinary Association for Intensive Care and Emergency Medicine (Deutsche Interdisziplinäre Vereinigung für Intensiv- und Notfallmedizin; DIVI), ICU nurse understaffing affects 45% and 74% of all units, respectively. In addition, in Germany, more than half of intensive care nurses work part-time, and many are considering leaving the profession [2]. The reasons are diverse but certainly include the unique circumstances in ICUs, which place high demands on health care professionals (HCPs), patients, and relatives—demands that worsen with increasing short-staffing. Exposure to adverse events, such as critical incidents in intensive care, can lead to secondary traumatization [3]. In a nonrepresentative survey conducted in 2013 among emergency nurses in Scotland, 75% of the sample reported experiencing 1 or more secondary traumatic stress symptoms in the previous week [4]. Additionally, potentially traumatizing events in the workplace are linked to depression, addiction, and suicidality, as stated in an annual report by the DIVI [5]. A significant number of HCPs in intensive care exhibit psychosomatic symptoms or signs of burnout [6].

Despite the negative impact on HCPs themselves, high stress also harms the health care system: nurses in poor physical and mental health report significantly more medical errors than those in better health [7]. As the connection between practitioners’ mental health and the quality of care is increasingly acknowledged [8], these circumstances raise important concerns about patient safety.

For patients and their relatives, the ICU is associated with significant stress and pressure. A systematic review of 42 prospective cross-sectional studies [9] identified stressors related to disease and treatment (eg, pain), mental health (eg, fear of death), communication (eg, inability to speak, lack of information about one’s condition), and the ICU environment (eg, noise, seeing, or hearing other patients). Patients are exposed to multiple potentially traumatic experiences during their treatment [10]. The observation that many patients experience new or worsening cognitive (eg, memory impairments), physical (eg, difficulties with activities of daily living), or psychological symptoms (eg, depression) after an ICU stay has led to the classification of postintensive care syndrome (PICS) [11,12]. In a prospective study conducted across 16 ICUs in Japan, 63.5% of participating survivors experienced at least one PICS-related impairment 6 months after their ICU stay [13]. Psychological symptoms related to the ICU stay can also be observed in relatives, such as anxiety, posttraumatic stress disorder, or depression, a condition referred to as postintensive care syndrome—family [11], which in some cases may be even more pronounced than in patients [13]. The German Association for Neurorehabilitation [14] has recommended implementing additional measures alongside standard ICU treatment to prevent PICS and to address existing PICS-related impairments. These measures target physical (eg, early mobilization), cognitive (eg, delirium prophylaxis), and psychological symptoms (eg, ICU diaries, improved communication, and family support). The stress experienced by ICU patients and their relatives may further intensify the emotional demands placed on HCPs, potentially exacerbating the issues described above and creating a downward spiral.

From a theoretical perspective, this phenomenon of accumulating stressors and problems can be explained by the Conservation of Resources (COR) theory [15]. The COR theory is an integrated, motivational theory of resources that focuses on coping and resource gain. It is based on the fundamental assumption that people tend to protect their existing resources and abilities while striving to acquire new ones [16]. According to this theory, stress is defined as a response to environmental conditions in which resource loss is threatened, has occurred, or cannot be offset by a gain in resources. The COR model particularly suggests that (threatened) resource losses can lead to further losses, reinforcing the downward spiral described above.

By contrast, also embedded in COR theory, positive circular processes—referred to as “positive gain spirals”—describe a salutogenetic process involving positive development and growth. The availability of key resources fosters the development and acquisition of additional resources: “resources cotravel in resource caravans” [17]. According to COR theory, the provision of additional resources, such as integrated psychosocial care, could therefore initiate a positive developmental spiral. COR theory has already been applied to perceived stress among nurses [18], the etiology of nurse burnout [19,20], and the development of posttraumatic stress disorder in survivors of COVID-19 [20]. While COR theory primarily focuses on resource losses and gains at the individual level, one study also highlights that resource loss in nurses, leading to burnout, should be addressed at the institutional level [19].

A broader perspective on organizational resources, demands, and their relationship to psychological and occupational health is offered by the concept of the Psychosocial Safety Climate (PSC) [21]. PSC refers to employees’ perceptions of the extent to which their organization considers their mental health in decision-making, particularly in the design of working conditions. In line with this, nurses who perceived their workplace as highly supportive of their well-being were twice as likely to report better physical health [7]. Interventions based on this concept target the root causes of stressful working conditions, rather than just addressing the stress symptoms of the employees themselves. One of its goals is to establish standards for best practices in stress prevention within organizations and to develop a solid evidence base for effective prevention and intervention strategies [22]. An increasing number of studies show that PSC is positively correlated with factors such as motivation, productivity, and self-reported health status, as well as a reduced number of workdays lost due to absence [23,24]. At the same time, PSC is negatively correlated with job and career change intentions [25].

Based on both evidence and theory, the need for psychosocial care as an intervention during and after intensive care is undisputed; however, it is being addressed only slowly [26,27]. Barriers remain significant: factors such as time constraints and lack of experience hinder the widespread use of such support [4]. Moreover, not only patients and their relatives, but also ICU HCPs, require access to psychosocial care, as has been advocated [28]. Recently, the Federal Joint Committee in Germany published a decision to amend the regulations for intensive care centers. It mandates “psychological support and crisis intervention for patients and staff to be available every working day” [29]. However, in the current care landscape of the German health care system, there are no integrative structures for the psychosocial care of ICU teams, patients, and their relatives. To our knowledge, corresponding interventions have never been systematically developed based on theory and evidence, nor with the involvement of all relevant stakeholder groups.

Initial experiences with an integrated psychosocial care concept have been gained in 2 project partner institutions, Charité Berlin and Ulm University Medical Center, where psychosocial care in ICUs was implemented during the COVID-19 pandemic [30] in response to the increased pressure on ICU HCPs, patients, and their relatives. Through an iterative and needs-focused approach, various psychosocial treatment tools, such as individual counseling, team supervision, health circles, and efficacy monitoring surveys, were selected, developed, and implemented for HCPs, patients, and their relatives. Specifically at Charité Berlin, up to 15 psychologists from the Psychosocial Emergency Care department worked as part of the ICU team (team-integrated), providing care for patients, relatives, and HCPs. Over 500 end-of-life care sessions were conducted. To date, 3 similar interventions have been carried out at the University of Ulm. While this makeshift intervention appears to have a positive effect, its design is based solely on clinical experience, and its feasibility and efficacy have yet to be empirically evaluated.

Effectively addressing patients, their relatives, and ICU HCPs with a single intervention would require the development of a complex intervention [31]. Complex interventions are characterized by several factors: they consist of multiple interacting components, target different groups and organizational levels simultaneously, expect a range of different outcomes, and involve a high degree of flexibility in design. The Medical Research Council (MRC) proposes a framework for developing and evaluating complex interventions, which prioritizes understanding both the contextual factors and the mechanisms of change, as they illustrate the connection between the intervention components and the outcome [32]. The development of a complex intervention is grounded in available evidence and theory, supplemented by participatory measures to incorporate the target group’s perspective.

### Objectives

This study aims to address the stress-related symptoms and consequences previously mentioned, primarily in ICU HCPs, as well as in patients and their relatives. The objective of the IPS-Pilot project (German: *Integrierte Psychosoziale Versorgung*; English: “Integrated Psychosocial Care”) is to (1) develop an integrated psychosocial care intervention (phase A), and then (2) assess the feasibility of its implementation and evaluation in a randomized controlled pilot trial in the ICU setting (phase B).

This protocol reports on phase A.

Our primary research question in this phase is “How should an integrated psychosocial care concept be designed to address the specific psychosocial needs of ICU HCPs, as well as those of intensive care patients and their relatives?”

The secondary research questions are (1) What are the psychosocial needs of ICU HCPs, ICU patients, and their relatives? and (2) What are the potential benefits and challenges of an integrated psychosocial intervention in the ICU?

Our focus is on identifying which needs (psychosocial and otherwise) must be addressed by the intervention to be developed, as well as the hurdles and resources important for its implementation. This will ensure the best possible practicability in the development of interventions, maximizing both effectiveness and acceptance by those affected.

## Methods

### Responsibilities

The IPS-Pilot project is coordinated by the Ulm University Medical Center (Department of Psychosomatic Medicine and Psychotherapy) in collaboration with the Otto von Guericke University Magdeburg (Institute of Social Medicine and Health Systems Research), the University Medical Centre Magdeburg A.ö.R. (Department of Psychosomatic Medicine and Psychotherapy), and Charité University Medicine Berlin (Department of Psychosomatic Medicine).

### Study Design

**Figure 1:**
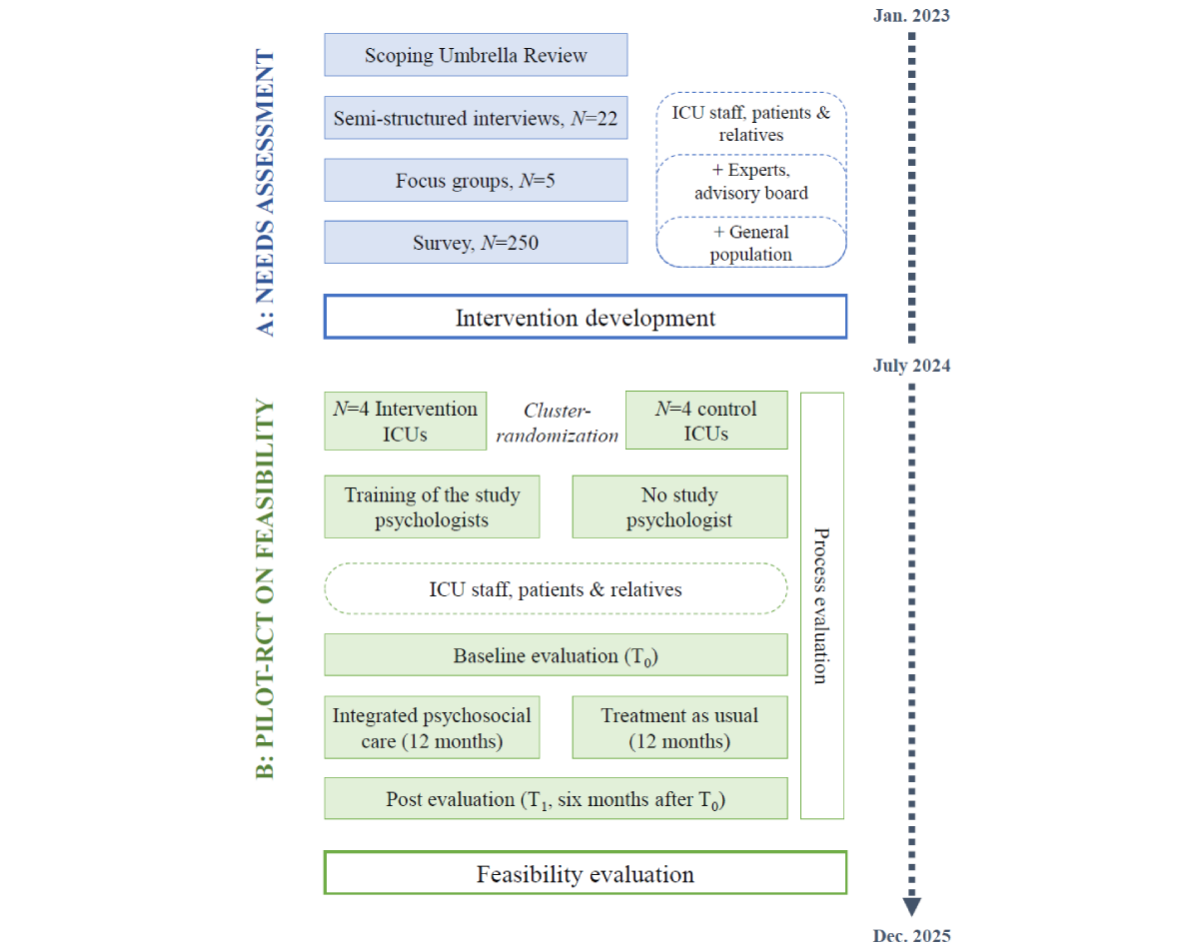
Overview on the study timeline. Timeline on the right side ranging from start to finish of the IPS-Pilot project and covering phase A and B. Rounded rectangles indicate sample origin. Blue boxes indicate substudies of the needs assessment and intervention development (phase A); green boxes indicate steps of the Pilot RCT on intervention feasibility in phase B which is to be covered in a separate study protocol.

The following protocol is based on the SPIRIT (Standard Protocol Items: Recommendations for Interventional Trials) guidelines [32] (see Multimedia Appendix 1) and the CONSORT (Consolidated Standards of Reporting Trials) statement [33]. IPS-Pilot is a mixed-methods study that will be conducted in 2 phases according to the MRC’s framework [31] for the development and evaluation of complex interventions; for reference, see Figure 1. The project runs from January 2023 to December 2025. This protocol focuses on the first study phase (phase A: needs assessment, intervention development, and optimization; January 2023 to June 2024), as the specific implementation and evaluation concept for feasibility in the second phase (phase B) will depend on the intervention developed in phase A. As shown in Figure 1, data collection for phase A and partial analyses for the intervention development were completed by June 2024, while the final analyses are still pending.

### Phase A: Gathering Evidence and Development of Intervention

#### Overview

In phase A, we assess the psychosocial needs of ICU patients, their relatives, and ICU HCPs through the evaluation of existing research (substudy 1: scoping umbrella review) and original data collection using qualitative (substudy 2) and quantitative (substudy 3) methods. The data from these studies will be used for intervention development in the fourth substudy.

#### Substudy 1: Scoping Umbrella Review

##### Methodological Overview

This substudy is registered online [34]. Numerous studies and reviews have examined the relevant needs, typically focusing on 1 of the 3 perspectives mentioned above. There are reviews addressing patient needs [35], the needs of relatives of ICU patients [36], and those of ICU HCPs [37]. However, to our knowledge, there is no comprehensive overview that encompasses all 3 perspectives. With the planned scoping umbrella review, we aim to systematically compile and compare studies on the needs and requirements of the various groups involved in ICU care. The findings can then be incorporated into the subsequent development of the intervention. This scoping umbrella review aims to provide an overview of systematically collected and evaluated reviews and meta-analyses addressing needs and demands in the ICU setting. The identified needs will be categorized, with a particular focus on the psychological and psychosocial needs of all those involved in the treatment process. This review will offer a comprehensive overview of the current state of research, and the insights gained can serve as a foundation for conceptualizing targeted psychosocial interventions in ICUs.

##### Inclusion and Exclusion Criteria

All systematic reviews or meta-analyses that summarize primary studies assessing the needs of individuals directly involved in ICU care, including patients, their physicians and nurses, and relatives, will be included. We will also consider reported factors that indicate a problem or suggest an underlying cause related to a specific need.

Studies focusing solely on pharmacological outcomes in neonatal or pediatric ICUs, as well as studies conducted exclusively in rehabilitation or palliative care settings, will be excluded.

##### Data Collection and Analysis

The search strategy includes both medical databases (MEDLINE via PubMed, Embase via Ovid, and Scopus) and social science databases (PsycINFO via EBSCOhost). In addition, we conducted searches in the Cochrane Database of Systematic Reviews and PROSPERO (International Prospective Register of Systematic Reviews), which are specifically dedicated to reviews. We are also performing a hand search of the reference lists of the included reviews. Titles and abstracts are being screened based on the research question, developed using the Person-Content-Context-Method framework of the Joanna Briggs Institute [38]. The review process follows the framework outlined by Levac et al [39]. Two researchers (WS and SP) independently screened and selected titles and abstracts. Full-text versions of potentially relevant articles were obtained, and inclusion and exclusion criteria were applied. Disagreements were resolved through discussion within the review team. A standardized data extraction form, developed by researchers and professionals with a focus on the usability of findings for intervention development, was used. The software ASReview (ASReview Team) was used for title and abstract screening, and EndNote (Clarivate Plc) was used to manage the literature database. Findings will be synthesized narratively and, if possible, quantitatively assessed and categorized. Categories will focus on study design, perspectives, and identified needs. Reporting will follow the guidelines outlined in PRISMA-ScR (Preferred Reporting Items for Systematic Reviews and Meta-Analyses Extension for Scoping Reviews) [40]. The PRISMA-ScR checklist and the search string are provided in Multimedia Appendix 2.

#### Substudy 2: Interviews and Focus Group Discussions

##### Methodological Overview

This substudy is registered online [41]. To evaluate ICU-related stress factors and needs regarding the intervention across all 3 target groups, individual interviews were conducted with former ICU patients, relatives of former ICU patients, and ICU HCPs in Germany. These insights were then explored further in focus groups, where HCPs, former patients, relatives, psychologists, and experts discussed the content requirements and implementation conditions for an integrated psychosocial intervention in ICUs. The research questions in this substudy were exploratory and therefore nondirectional. The primary research question aimed to identify psychosocial needs in intensive care from the perspectives of patients, relatives, and HCPs. Secondary research questions focused on exploring support factors and barriers to the use of an integrated psychosocial intervention, as well as design requirements to make such an intervention accessible and acceptable to those affected. The goal was not to identify converging perspectives, but rather to gather diverse viewpoints to develop a vivid and comprehensive understanding of the phenomenon of interest.

##### Sample and Eligibility Criteria

We conducted interviews with ICU HCPs, former ICU patients, and relatives of former ICU patients, as well as focus group discussions, each involving up to 6 participants. Participants either belonged to 1 of the 3 aforementioned groups or had relevant professional experience, such as working as an ICU-integrated psychologist or holding administrative roles (eg, controlling, clinic directors). We aimed to include patients, relatives, and HCPs with and without prior experience of interacting with an ICU-integrated ward psychologist. Study participants had to be at least 18 years old and provide informed written consent to the scientific staff conducting the interviews. Only patients and relatives who had received treatment in the participating ICUs (Berlin, Magdeburg, and Ulm) within the last 6 months were included. ICU HCPs with varying levels of work experience and professional backgrounds (nurses and doctors from different hierarchies) were also included. Participants were recruited by directly approaching them in the context of their treatment or occupation in the ICUs of the participating hospitals.

##### Data Collection

Before data collection, a test interview was conducted. The interviews and focus group discussions were carried out either face-to-face or online via data protection–compliant videoconferencing systems at the 3 study sites in Germany. These sessions were conducted by project staff trained in qualitative data collection. All interviews and discussions were audio-recorded using a recording device and transcribed by a qualified external contractor. Semistructured questions were developed for conducting the interviews and focus group discussions. The guiding questions were adapted during data collection as needed. For both the interviews and the focus group discussions, sociodemographic data (eg, age, gender, work position, and experience in ICU) were collected to better categorize the results after each session. These data were gathered using a standardized questionnaire filled out under a pseudonym. The interview and focus group guides can be found in Multimedia Appendix 3.

##### Data Analysis

Depending on the research questions, the data material is analyzed using qualitative content analysis [42] with the aid of MAXQDA (VERBI GmbH). For the interviews, an initial code system was developed deductively, based on relevant literature [42] and previously identified topics. This code system was then expanded inductively during the analysis of the first few interviews (5/22, 23%). The remaining material was coded by pairs of independent researchers (KS, HH, GK, and RKN) and validated through consensus. If any differences could not be resolved through discussion, the results were presented to a third team member to reach a consensus. The same procedure will be applied to the focus group discussions, with the category system based on the final code system used for the interviews. We aim to ensure a methodologically controlled analysis process and high-quality data analysis through communicative, interdisciplinary validation within the research team.

#### Substudy 3: Survey

##### Quantifying Psychosocial Needs and Assessing Care Model Implementation

This study is registered online [43]. In substudy 3, an online questionnaire survey was conducted to complement the data on needs collected in substudies 1 and 2. The goal was to quantify the aforementioned needs. Additionally, the survey gathered information on known and utilized care models, including their frequency and nature of use, as well as obstacles encountered in implementing a potential intervention.

##### Sample and Eligibility Criteria

The target sample size was 250 participants. Participants had to be of legal age, able to complete the questionnaire, and provide informed consent. The substudy aimed to quantify and compare psychosocial needs across 4 groups: former ICU patients, relatives of former ICU patients, ICU HCPs, and the general population. We planned to recruit at least 50 participants in each of the 4 groups. Participation in the survey was anonymous and voluntary.

##### Data Collection

**Figure 2:**
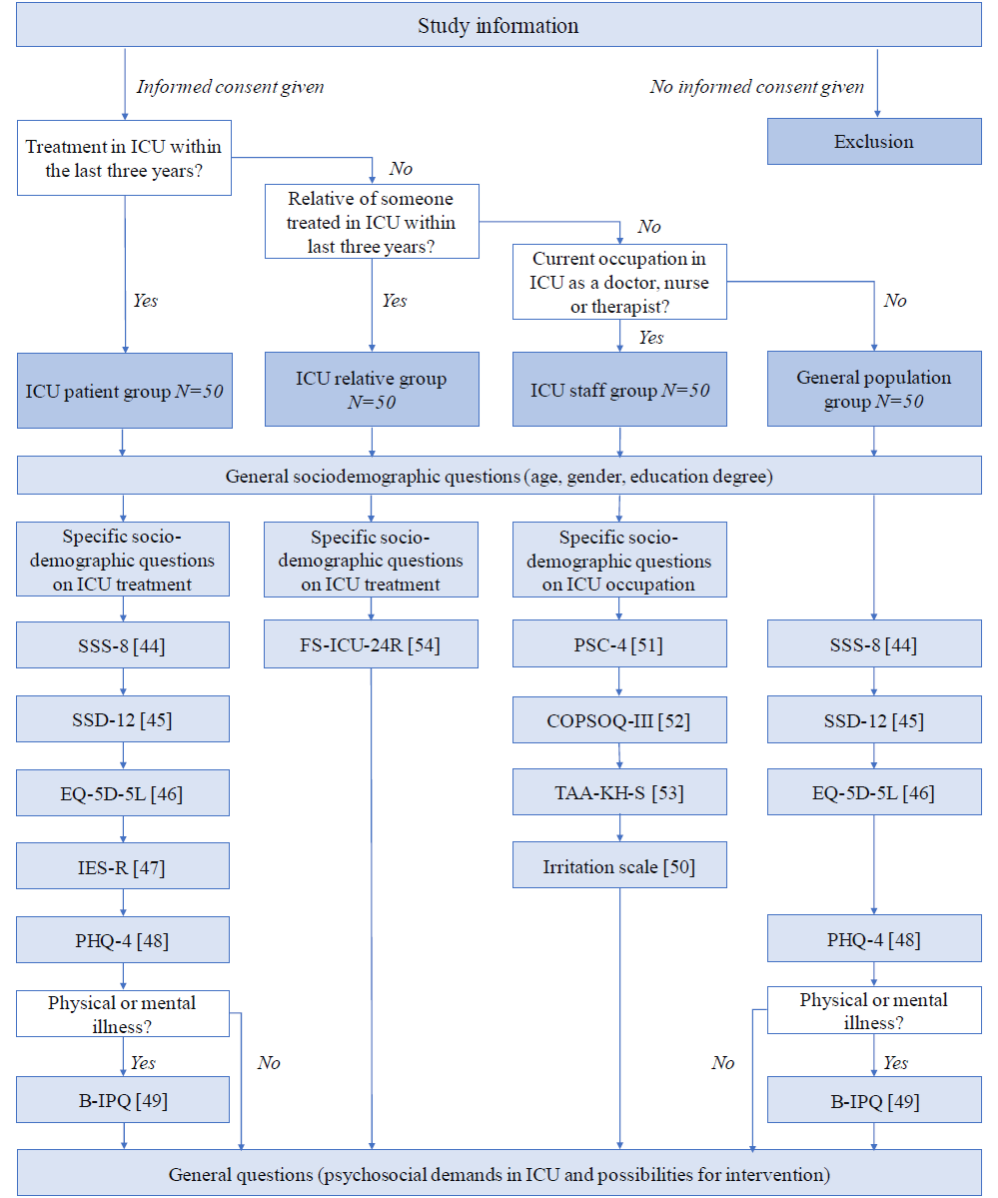
Overview on the administered scales [44-54] in the questionnaire depending on group allocation by filter questions.

Four different sets of partially overlapping questionnaires were developed, each tailored to 1 of the 4 target groups. Participants were presented with a combination of individually developed questions and items from validated questionnaires, depending on the group to which they belonged. Participants were allocated to 1 of the 4 groups described above through consecutive filter questions, which determined whether they were an ICU patient within the last 3 years, a relative of a former ICU patient, an HCP currently working in the ICU, or none of the above. Figure 2 illustrates the allocation procedure and the questionnaires administered to each group (also see [44-54]).

The survey was conducted and evaluated digitally and anonymously using LimeSurvey (LimeSurvey GmbH) online [55], with recruitment through German professional societies, interest groups, and other relevant channels. A pretest with 20 participants was conducted to assess the comprehensibility and feasibility of the survey.

##### Data Analysis

We will use descriptive statistics to analyze and report the findings, focusing on differences in psychosocial needs and support across participant groups. As all analyses are exploratory, no hypotheses were specified. Exploratory analyses may involve examining patterns of psychosocial needs and support across demographic variables. Missing data will be addressed by reporting the available data without imputation.

#### Substudy 4: Intervention Development and Optimization Process

**Figure 3:**
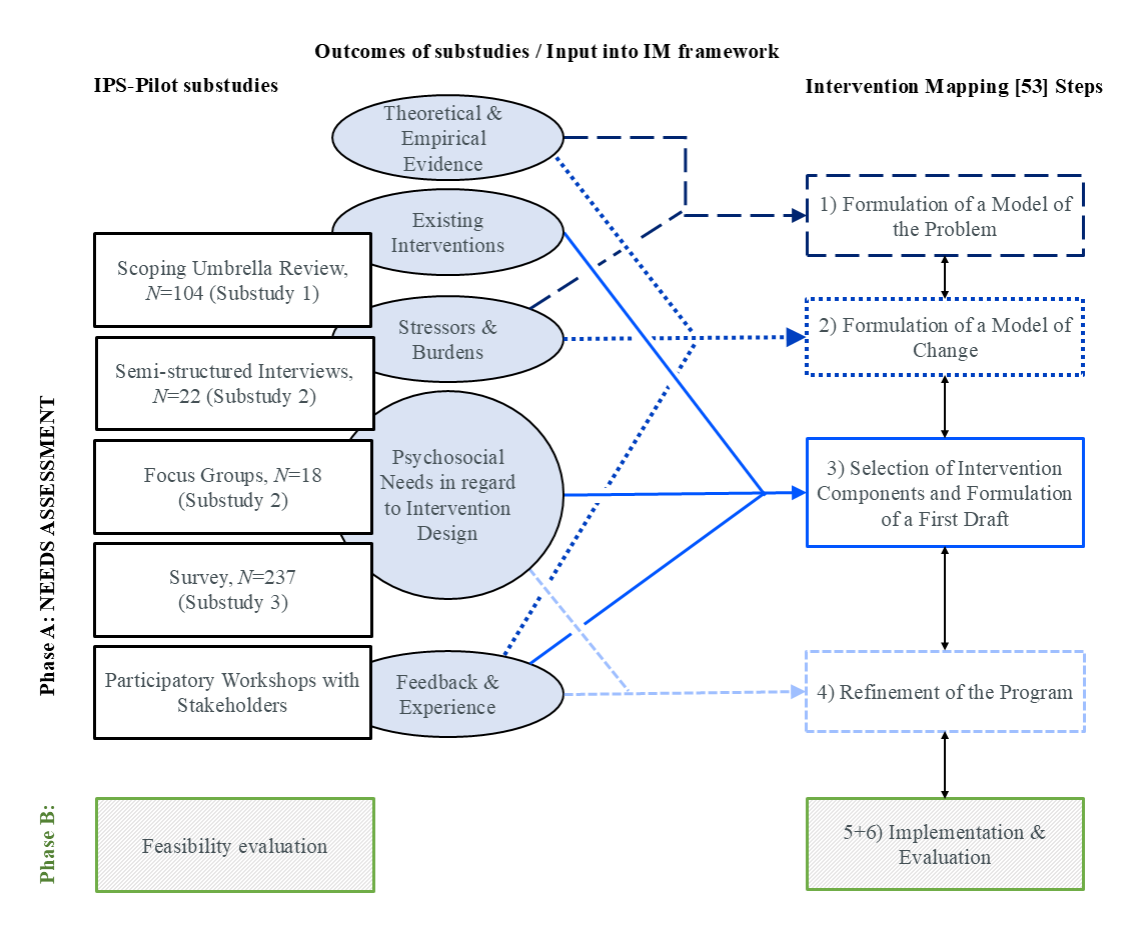
Synthesis and integration process of the sub-studies (left column) and their respective outcomes as well as theoretical and empirical input (middle column) into the steps of the IM process (right column).

For the intervention development, we planned an extensive multistep participatory process, structured according to the MRC framework [31,56]. The specific steps follow the Intervention Mapping (IM) framework [56]. To ensure a comprehensive understanding of the needs of our target groups, the evidence obtained from substudies 1, 2, and 3 was summarized and integrated. Figure 3 provides an overview of the synthesis process. For example, in step 3 of the IM framework, effective interventions in ICUs identified in the scoping umbrella review (Substudy 1) were listed, organized, discussed, and selected by the research team based on their potential feasibility in German ICUs. In the context of 1 of 3 workshops, this selection of intervention tools was compared and supplemented with findings from the interviews and focus groups regarding desired interventions (substudy 2). The evidence was then aligned with and incorporated into our model of change, synthesizing it with our theoretical approach—the resource-oriented perspective of the PSC [21] and COR [16] theory. Following this, a first draft of the intervention was developed according to the IM concept [56], within the scientific study team and in close collaboration with experts. During this process, 3 online and face-to-face workshops were held, involving members of the study team and stakeholders (former ICU patients, relatives, and various representatives of HCPs, such as physicians, nurses, and therapists). Stakeholders were selected for participation and approached personally by the study team, for example, as members of the IPS-Pilot advisory board. The goals of these workshops were to (1) develop the logic model of change, mapping the interrelationships and corresponding intervention components (IM step 2); (2) select intervention components that had already been evaluated in previous studies (substudy 1) or suggested by target groups (substudy 2; IM step 3); and (3) review and refine the intervention draft, as well as the planned implementation process, with the stakeholders (IM step 4). For this substep, methods from the design thinking approach [57] were used, specifically the Disney Strategy [58], which has been applied in various program and intervention design processes, including those involving stakeholder participation [59,60]. Discussion topics included the description, timing, scope, and other framework conditions of the intervention components. The final draft of the intervention tools was then refined through the survey (substudy 3), in which the needs were quantified. The resulting psychosocial care intervention is primarily designed to be delivered by a clinically experienced psychologist (in psychotherapeutic training; hereinafter referred to as the IPS therapist) with a 50% deputization. This IPS therapist will be an integral part of the permanent ICU HCP team for the duration of the intervention. Individual components of the complex intervention may be delivered by other HCPs, depending on local availability. The final concept (hereinafter referred to as the IPS intervention) will be documented in the form of an intervention protocol and an intervention manual, which will provide instructions for the interventions to be administered by the IPS therapist.

#### Patient and Public Involvement

In all substudies, the perspectives of our target groups—ICU HCPs, patients, and their relatives—are collected and incorporated. In substudy 1, their needs are included indirectly through research on existing studies. In substudies 2 and 3, they are directly involved as participants in the interviews, focus groups, and survey. In our fourth and final substudy, we invited all target groups to participate in workshops where the concrete intervention design was discussed. Throughout the project, we will continue to engage our target groups in our advisory board.

### Outlook on Phase B: Pilot Study

The feasibility of a pilot RCT on the IPS intervention developed in phase A will be evaluated in a multicenter study involving the 3 sites in Germany. Eight nonspecialized ICUs for adults will be selected from the university hospitals in Berlin, Magdeburg, and Ulm. Using a randomized cluster design, 4 ICUs will be assigned to the intervention group, and 4 to the control group. Over the course of 12 months, psychologists in psychotherapeutic training will be assigned to each of the 4 intervention ICUs as IPS therapists from mid-2024 to mid-2025. Meanwhile, a survey with 2 measurement points will be conducted across all participating wards, involving HCPs, patients, and relatives, to evaluate feasibility criteria such as participation rate and practicability. A separate study protocol for phase B will be submitted and preregistered. Further descriptions of the methods and end points can be found in this study protocol.

### Ethics and Dissemination

With regard to scientific quality, the guidelines for ensuring good scientific practice from the German Research Foundation, as well as Good Epidemiological Practice [31], were followed.

Research ethics approval was obtained from the Ethics Committee of the University of Ulm (approval number 108/23-FSt/Sta) and the University Medical Centre Magdeburg (approval number 34/23), both of which were recognized as valid by Charité-Universitätsmedizin Berlin in accordance with §15 Section 2 of the Professional Code of Conduct of the Berlin Medical Association [61]. Any modifications to the study plan, as described above, were communicated to the ethics committees and the funder, and permission for the modifications was obtained where necessary.

The EU General Data Protection Regulation and the Federal Data Protection Act apply to data protection. A data protection concept has been developed in accordance with these guidelines, specifically addressing the planned methods of data collection, storage, and analysis. This concept has been approved by the data protection officers at Ulm University Medical Centre. Guidance on the procedure was obtained from the Center for Clinical Trials Ulm, and processing activities have been registered at all sites. A trustee office has been established to ensure the secure transfer of data. Personally identifiable data were only collected when necessary (eg, for data withdrawal after participation). These data were transferred solely between the involved parties using secure messaging systems. Pseudonyms or personal data were never transmitted via an unencrypted internet connection or any other medium.

The trial was conducted in accordance with the Declaration of Helsinki. Informed written consent was obtained from all participants (see Multimedia Appendix 4). Participants were informed that their participation was voluntary and could be discontinued or canceled at any time, before, during, or after participation. No payment was provided for participation.

## Results

The development of the IPS intervention was completed by the beginning of June 2024. Because of the time frame, analyses of the substudies were only partially completed at this stage, with a focus on psychosocial stressors and needs to be included in the intervention design. Through the 4 substudies, we aimed to establish both a logical model of the problem and a logical model of change. Together, these models describe the psychosocial needs of ICU patients, their relatives, and ICU HCPs, and explain how the stress caused by these needs in ICUs could be addressed and effectively reduced through a complex psychosocial intervention. To formulate these models, we collected, compared, classified, and quantified the demands and needs from the 3 different perspectives, and will continue to analyze them. The final results of the individual substudies will be reported separately in 2025.

Data collection and analysis for the scoping umbrella review (substudy 1) are yet to be completed, due to the breadth of the included studies. A total of 109 studies on psychosocial needs, as well as existing psychosocial interventions, were included in the synthesis for intervention development. In the next step, the included studies will be analyzed in more detail with regard to psychosocial needs and requirements. The final analysis of the scoping umbrella review will be completed in 2025.

Interviews and focus groups (substudy 2) were completed in November 2023 and March 2024, respectively. We conducted interviews with 10 ICU HCPs, 6 former ICU patients, and 6 relatives of former ICU patients, as well as 5 focus group discussions with a total of 18 participants. Analysis of the interview data was completed in August 2024; analysis of the focus groups is expected to be completed by April 2025. The survey (substudy 3) was conducted from April to June 2024 and collected data from 51 ICU patients, 52 relatives, 73 ICU HCPs, and 61 participants from the general population. Analysis of the data was completed in October 2024. The workshops in substudy 4 were conducted in March (n=11 participants), April (n=15 participants), and May 2024 (n=20 participants). The intervention manual was completed at the beginning of June 2024.

## Discussion

This study protocol provides an overview of a structured, theory-guided, multimethod, participatory approach for designing an integrated psychosocial intervention in ICUs within the framework of the IPS-Pilot project. The methods described in the protocol are expected to lead to a deeper understanding of the psychosocial needs of our target groups and, therefore, ensure the best possible fit of the resulting IPS intervention.

During the COVID-19 pandemic, public awareness of the psychosocial burdens and needs of HCPs, patients, and relatives in the ICU context increased. To address the growing pressure on these groups, a makeshift intervention—in the form of a psychologist integrated into the ICU team—was implemented at Berlin Charité and Ulm University Medical Center. Our experiences during the pandemic indicated that all groups benefited from this complex psychosocial treatment. However, there is still a lack of scientifically grounded evidence regarding the psychosocial needs that should be addressed through a complex intervention in the ICU context—along with its optimal design, feasibility, and, ultimately, its effectiveness.

This study protocol aims to make the development process of the IPS intervention transparent and accessible. A key limitation arises from the complexity of the development process itself. As all steps outlined in the IM framework [56] required a synthesis of theory alongside existing and newly generated evidence, the procedures and analyses of our interdependent substudies were constrained by a tight time frame. This time frame may seem at odds with intervention development frameworks, which emphasize a dynamic rather than linear development process [31,56]. Therefore, to avoid delays and ensure sufficient knowledge synthesis, intermediate or partial data analyses and repeated feedback loops between substudies were necessary. This required adequate time resources and close collaboration within the scientific team.

It is well established that HCPs experience a high stress burden, which also affects patients and their relatives—albeit in different ways. Based on interacting theoretical concepts of individual [15,16] and structural stress [17,21], the stress perceived by these 3 groups appears to be interconnected. In addition to the possible interdependence of the 3 groups, there are differences in their perception of various stressors, such as the perceived importance of post-ICU outcomes (eg, survival rate). This highlights the need for an intervention that includes the perspectives of patients, relatives, and HCPs, and mediates between them [62]. There is empirical evidence on the efficacy of intra-ICU psychological care, such as for reducing post-ICU depression, posttraumatic stress disorder, and anxiety [63,64]. However, all controlled trials on stress-reduction interventions in ICUs known to us have focused solely on parts of these groups, such as patients [65], relatives [66], or HCPs [67]. To our knowledge, the IPS-Pilot is the first project to systematically assess psychosocial needs in ICUs and to develop and evaluate a complex intervention that addresses these needs for all involved groups at once, using a participatory process grounded in theory and evidence.

It remains to be examined whether integrated and simultaneous psychosocial care for these groups is beneficial or may lead to difficulties or even adverse effects. Based on prior experiences in Berlin and Ulm, the integration of a psychologist into ICU teams appears to be accepted, perceived as helpful, and frequently utilized by HCPs, patients, and their relatives. The integration of psychological care for both patients and professionals in the ICU context has also been advocated by professional associations, legal committees, and, based on our experience, by the involved groups themselves (eg, staff members).

A nonexperimental evaluation of patient and relative surveys conducted in Brazil [68] highlighted the different roles of psychologists working in ICUs. ICU psychologists can help patients and their relatives manage stress and negative emotions experienced during ICU treatment. In addition, psychosocial care may support HCPs in managing both individual and work-induced stress. The authors conclude that psychologists integrated into ICU teams can help improve team relationships and communication among HCPs, patients, and their relatives. A review on the role of psychologists in pediatric ICUs in Britain suggested that team integration and the mere presence of a psychologist may lead to greater consideration of psychological factors by the ICU team. Additionally, the ICU psychologist’s easy approachability allows HCPs to seek advice on personally demanding cases or request short debriefing sessions [69].

By contrast, one could argue that consulting the ICU team or individual team members while treating patients and their families as part of the interdisciplinary team could lead to role conflict and affect the professional independence of the psychologist. Team members may be hesitant to seek advice from a team-integrated psychologist due to an unclear understanding of the psychologist’s role or concerns about confidentiality or stigmatization, as research on the acceptability of workplace-integrated psychotherapeutic consultation suggests [70]. Similar difficulties may arise if supervision is needed due to a team conflict. Additionally, HCPs might fear being seen seeking the psychologist’s advice, a limiting factor previously identified in studies on the use of psychological consultation in the workplace [71]. By involving stakeholders at multiple points throughout our intervention development process, we aimed to identify these and other potential issues in the intervention’s implementation and explore possible solutions, such as outsourcing conflict-laden, multiperson interventions to external supporters. Based on the experiences of the research team and insights gained from former ICU ward psychologists in the focus groups, we will also include measures to support IPS therapists during the evaluation phase, such as supervision and intervision. The evidence described above clearly highlights the importance of a careful, trusting approach with a high level of confidentiality, as well as the need to consider possible role conflicts and the psychologist’s role itself. These factors, which may limit the feasibility of the IPS intervention, were considered during the development process and will also be surveyed and evaluated in the feasibility study, which will be described in detail in the study protocol for phase B of the IPS-Pilot project.

In conclusion, there is a lack of systematically gathered evidence on how a complex, team-integrated psychosocial intervention in ICUs should be ideally designed, and whether the outcome of such an intervention design process proves to be feasible and effective. Through a multimethod approach, we aim to gain a broader insight into the needs of our target groups. Difficulties encountered in phase A, resulting from our choice of methods and targeted samples, will be monitored and included in the feasibility analysis during phase B.

Through a feasibility trial, the conditions that contribute to the successful implementation of the intervention and enable the execution of its mechanisms of change can be explored. Based on the results of phase B, it will then be determined whether a randomized controlled trial on the efficacy of the IPS intervention is feasible and should be conducted. Although our study is conducted solely in Germany to assess feasibility in this specific health care context, the IPS intervention may also be applicable in other health care settings. The similarity of the IPS intervention to examples from countries such as Brazil [68] and the United Kingdom [69] highlights the international interest and relevance of psychosocial support in ICUs. Furthermore, because the development of the IPS intervention is based not only on a needs assessment of German samples but also on international evidence gathered in the scoping umbrella review and internationally applied stress theories [17-24], there is reason to believe in its generalizability and potential for international feasibility.

## Appendix

Multimedia Appendix 1 SPIRIT checklist.

Multimedia Appendix 2 PRISMA-ScR checklist and search string of the Scoping Umbrella Review.

Multimedia Appendix 3 Interview and Focus Group guides.

Multimedia Appendix 4 Sample Participant Information and Consent form.

## Abbreviations

CONSORT: Consolidated Standards of Reporting Trials
COR: Conservation of Resources
DIVI: German Interdisciplinary Association for Intensive Care and Emergency Medicine (German: Deutsche Interdisziplinäre Vereinigung für Intensiv- und Notfallmedizin)
HCP: health care professional
ICU: intensive care unit
IM: Intervention Mapping
IPS: Integrated Psychosocial Care (German: Integrierte Psychosoziale Versorgung)
MRC: Medical Research Council
PICS: postintensive care syndrome
PRISMA-ScR: Preferred Reporting Items for Systematic Reviews and Meta-Analyses Extension for Scoping Reviews
PROSPERO: International Prospective Register of Systematic Reviews
PSC: Psychosocial Safety Climate
SPIRIT: Standard Protocol Items: Recommendations for Interventional Trials

## Data Availability

The data sets generated or analyzed during this study are available from the corresponding author (SFN) or principal investigators (HG, CA, FJ, and MR) upon reasonable request. Participants can assign themselves to a mailing list if they are interested in updates on and results of the study. The results of the substudies will be made public via scientific publications and presentations at conferences for ICU HCPs and in the field of health care research in general. Deidentified individual clinical trial participant-level data cannot be shared due to strict ethics committee and staff council regulations.
