# Supplementary material for: Integrated Psychosocial Care in Intensive Care (IPS-Pilot): Protocol for the Systematic, Multimethod Development of a Complex Intervention (Phase A)
Source: JMIR Res Protoc. 2025 Jun 6;14:e65682. doi: 10.2196/65682 (PMC12181753; doi:10.2196/65682)
Supplement: Multimedia Appendix 2 [file resprot_v14i1e65682_app2.pdf]

Appendix 2: PRISMA-ScR Checklist and Search String for the Scoping Umbrella Review

Preferred Reporting Items for Systematic reviews and Meta-Analyses extension for Scoping Reviews (PRISMA-ScR) Checklist

SECTION	ITEM	PRISMA-ScR CHECKLIST ITEM	REPORTED ON PAGE #
**TITLE**			
Title	1	Identify the report as a scoping review.	N/A
**ABSTRACT**			
Structured summary	2	Provide a structured summary that includes (as applicable): background, objectives, eligibility criteria, sources of evidence, charting methods, results, and conclusions that relate to the review questions and objectives.	Abstract
**INTRODUCTION**			
Rationale	3	Describe the rationale for the review in the context of what is already known. Explain why the review questions/objectives lend themselves to a scoping review approach.	Substudy 1: Scoping Umbrella Review
Objectives	4	Provide an explicit statement of the questions and objectives being addressed with reference to their key elements (e.g., population or participants, concepts, and context) or other relevant key elements used to conceptualize the review questions and/or objectives.	Substudy 1: Scoping Umbrella Review
**METHODS**			
Protocol and registration	5	Indicate whether a review protocol exists; state if and where it can be accessed (e.g., a Web address); and if available, provide registration information, including the registration number.	Substudy 1: Scoping Umbrella Review
Eligibility criteria	6	Specify characteristics of the sources of evidence used as eligibility criteria (e.g., years considered, language, and publication status), and provide a rationale.	Substudy 1: Scoping Umbrella Review – Inclusion and exclusion criteria
Information sources*	7	Describe all information sources in the search (e.g., databases with dates of coverage and contact with authors to identify additional sources), as well as the date the most recent search was executed.	Substudy 1: Scoping Umbrella Review – Data collection and analysis
Search	8	Present the full electronic search strategy for at least 1 database, including any limits used, such that it could be repeated.	See below
Selection of sources of evidence†	9	State the process for selecting sources of evidence (i.e., screening and eligibility) included in the scoping review.	Substudy 1: Scoping Umbrella Review – Data collection and analysis
Data charting process‡	10	Describe the methods of charting data from the included sources of evidence (e.g., calibrated forms or forms that have been tested by the team before their use, and whether data charting was done independently or in duplicate) and any	Substudy 1: Scoping Umbrella Review – Data collection and analysis

SECTION	ITEM	PRISMA-ScR CHECKLIST ITEM	REPORTED ON PAGE #
		processes for obtaining and confirming data from investigators.	
Data items	11	List and define all variables for which data were sought and any assumptions and simplifications made.	Defined in registration: https://osf.io/cb67a
Critical appraisal of individual sources of evidence§	12	If done, provide a rationale for conducting a critical appraisal of included sources of evidence; describe the methods used and how this information was used in any data synthesis (if appropriate).	N/A
Synthesis of results	13	Describe the methods of handling and summarizing the data that were charted.	Substudy 1: Scoping Umbrella Review
**RESULTS**			
Selection of sources of evidence	14	Give numbers of sources of evidence screened, assessed for eligibility, and included in the review, with reasons for exclusions at each stage, ideally using a flow diagram.	N/A
Characteristics of sources of evidence	15	For each source of evidence, present characteristics for which data were charted and provide the citations.	N/A
Critical appraisal within sources of evidence	16	If done, present data on critical appraisal of included sources of evidence (see item 12).	N/A
Results of individual sources of evidence	17	For each included source of evidence, present the relevant data that were charted that relate to the review questions and objectives.	N/A
Synthesis of results	18	Summarize and/or present the charting results as they relate to the review questions and objectives.	N/A
**DISCUSSION**			
Summary of evidence	19	Summarize the main results (including an overview of concepts, themes, and types of evidence available), link to the review questions and objectives, and consider the relevance to key groups.	N/A
Limitations	20	Discuss the limitations of the scoping review process.	N/A
Conclusions	21	Provide a general interpretation of the results with respect to the review questions and objectives, as well as potential implications and/or next steps.	N/A
**FUNDING**			
Funding	22	Describe sources of funding for the included sources of evidence, as well as sources of funding for the scoping review. Describe the role of the funders of the scoping review.	Authors' contributions

JBI = Joanna Briggs Institute; PRISMA-ScR = Preferred Reporting Items for Systematic reviews and Meta-Analyses extension for Scoping Reviews.
* Where *sources of evidence* (see second footnote) are compiled from, such as bibliographic databases, social media platforms, and Web sites.

Appendix 2: PRISMA-ScR Checklist and Search String for the Scoping Umbrella Review

† A more inclusive/heterogeneous term used to account for the different types of evidence or data sources (e.g., quantitative and/or qualitative research, expert opinion, and policy documents) that may be eligible in a scoping review as opposed to only studies. This is not to be confused with *information sources* (see first footnote).
‡ The frameworks by Arksey and O'Malley (6) and Levac and colleagues (7) and the JBI guidance (4, 5) refer to the process of data extraction in a scoping review as data charting.
§ The process of systematically examining research evidence to assess its validity, results, and relevance before using it to inform a decision. This term is used for items 12 and 19 instead of "risk of bias" (which is more applicable to systematic reviews of interventions) to include and acknowledge the various sources of evidence that may be used in a scoping review (e.g., quantitative and/or qualitative research, expert opinion, and policy document).

Appendix 2: PRISMA-ScR Checklist and Search String for the Scoping Umbrella Review

Substudy 1: Scoping Umbrella Review – Search String

('demand':ab OR 'perspective':ab OR 'experience':ab OR 'ethics':ab OR satisfaction:ab OR 'job satisfaction':ab OR 'needs assessment':ab OR needs:ab OR empowerment:ab OR safety:ab OR emotion:ab OR 'expectation':ab OR 'behavior':ab OR 'social medicine':ab OR hrqol:ab OR 'health related quality of life':ab OR 'mental stress':ab OR 'social psychology':ab OR 'psychosocial':ab OR 'anxiety':ab OR ptbs:ab OR ptbd:ab OR delir:ab OR communication:ab OR 'strain':ab OR 'depression':ab OR 'psychological distress':ab OR engagement:ab OR 'sickness absent*':ab OR 'burnout':ab OR 'posttraumatic stress disorder':ab OR 'burden':ab) AND ('intensive care unit':ab OR icu:ab OR 'intensive medicine':ab) AND ('nursing':ab OR 'nurse':ab OR 'physician':ab OR 'doctor nurse relation':ab OR 'family':ab OR 'patient':ab OR 'therapist':ab OR 'physiotherapist':ab OR 'speech pathologist':ab OR 'occupational therapist':ab OR 'nursing staff':ab OR 'health care personnel':ab OR 'medical staff':ab OR 'teamwork':ab OR 'rapid response team':ab OR 'team nursing':ab OR 'anesthesiologist':ab OR 'doctor patient relationship':ab OR 'hospital patient':ab OR relatives:ab OR 'family centered care':ab OR 'peer group':ab OR peers:ab) NOT ('sedation':ab OR neonatal:ab OR 'pediatrics':ab OR 'neuromuscular electrical stimulation':ab OR 'melatonin':ab OR 'antibiotic':ab OR 'pressure injury':ab OR 'decubitus':ab) AND ([embase]/lim OR [medline]/lim OR [preprint]/lim) AND ([cochrane review]/lim OR [systematic review]/lim OR [meta analysis]/lim) AND ([embase]/lim OR [medline]/lim OR [preprint]/lim OR [pubmed-not-medline]/lim)
