# Supplementary material for: Integrated Psychosocial Care in Intensive Care (IPS-Pilot): Protocol for the Systematic, Multimethod Development of a Complex Intervention (Phase A)
Source: JMIR Res Protoc. 2025 Jun 6;14:e65682. doi: 10.2196/65682 (PMC12181753; doi:10.2196/65682)
Supplement: Multimedia Appendix 3 [file resprot_v14i1e65682_app3.pdf]

Integrated Psychosocial Care in Intensive Care (IPS-Pilot): Study Protocol of the Systematic, Multi-Method Development of a Complex Intervention (Phase A)

Appendix 4: Interview and Focus Group guides for Substudy 2

Contents

1. Interview guide for ICU HCPs
2. Interview guide for former ICU patients
3. Interview guide for relatives of former ICU patients
4. Focus group guide for former ward psychologists
5. Focus group guide for HCPs with experience in working with a ward psychologist
6. Focus group guide for HCPs without experience in working with a ward psychologist
7. Focus group guide for former ICU patients and relatives
8. Focus group guide for participants with expertise in clinical structures

Appendix 3: Interview and Focus Group guides for Substudy 2

Interview guide for HCPs

Leitfaden Interview für Mitarbeitende

Herzlich willkommen und vielen Dank für Ihre Bereitschaft zur Teilnahme.

Mein Name ist: ______________________________

Vorstellung Moderator*in

Gemeinsam mit der Universität Ulm, der Universität Magdeburg und der Charité Berlin führen wir eine Studie in dem Forschungsprojekt „IPS-Pilot: Integrierte psychosoziale Unterstützung in der Intensivmedizin“ durch. Zu diesem Thema möchte ich Ihnen heute einige Fragen stellen, bei denen es um Ihre Erfahrungen und Einschätzungen geht.

Im Rahmen der COVID-19-Pandemie sind auf einigen Intensivstationen in Deutschland Psycholog*innen in die intensivmedizinischen Teams integriert worden, um auf den Stationen mitzuarbeiten. Darauf möchten wir aufbauen und ein Konzept entwickeln, welches langfristig die psychosoziale Versorgung auf Intensivstationen betrifft. Das heutige Interview findet statt, damit wir von Ihren Erfahrungen und Ihrem Expert*innenwissen lernen können.

Dafür sind 30 bis maximal 60 Minuten eingeplant. Ich werde Sie nun über die Studie und die Datenschutzbestimmungen aufklären.

- Spätere Verwendung der Daten
- Angebot die Ergebnisse zu schicken auf Aufforderung an ips.pilot@uniklinik-ulm.de
- Form der Dokumentation (Tonband, Transkription, Pseudonymisierung)

Das Tonband ist jetzt eingeschaltet. Würden Sie mir bitte nochmals bestätigten, dass Sie mit der Aufzeichnung des Gesprächs auf Tonband einverstanden sind, dass ich Sie über den Datenschutz aufgeklärt habe und dass alle Angaben freiwillig sind?
Unser Interview besteht aus 4 Leitfragen, zu denen wir manchmal mit vertiefenden Fragen etwas ausführlicher in die Thematik einsteigen werden. Im ersten Teil des Gesprächs wird es um Belastungen im Arbeitsalltag gehen und in der zweiten Hälfte um Ihren Umgang mit denselben.
Wir beginnen das Interview jetzt.

Appendix 3: Interview and Focus Group guides for Substudy 2

Interview guide for HCPs

Aktuelle Situation und aktuelle Probleme (Anforderungen, Job Demands, Ressourcenverlust)

Die erste Frage stützt sich auf die Beobachtung, dass Mitarbeitende auf einer Intensivstation häufig mit besonderen Belastungen konfrontiert werden.

1: Was erleben Sie als belastend in Ihrer alltäglichen Arbeit?

Können Sie Beispiele nennen?
Was belastet Sie daran besonders?
Wie wirkt sich das auf Sie aus?
Wie wirkt sich das auf Ihr Team aus?
Woran merken Sie das?
10 Minuten

Aktuelle Ressourcen (Ressourcen, Organisation)

2. Gibt es in Ihrer Einrichtung Angebote zur psychosozialen Unterstützung? Welche?

Welche Erfahrungen haben Sie damit gemacht?
Könnte man hiervon auch etwas auf Ihrer Station anbieten?
Wie ist Ihrer Ansicht nach die Einstellung <u>Ihrer Vorgesetzten</u> zum Thema „psychosoziale Unterstützung"?
Wie ist Ihrer Ansicht nach die Einstellung <u>Ihrer Klinik</u> zum Thema „psychosoziale Unterstützung"?
10 Minuten

Appendix 3: Interview and Focus Group guides for Substudy 2
Interview guide for HCPs

Aktuelle Ressourcen (Ressourcen, Individuum)

3. Was hilft Ihnen derzeit, gut mit den Belastungen umzugehen?

Können Sie Beispiele nennen? (Kolleg*innen, private Ressourcen, Bewertung, Verhalten)

→ Mitarbeitende OHNE ITS-Psychologin:

Gibt es Situationen, in denen Sie sich eine Psychologin gewünscht hätten?
Können Sie Beispiele nennen?
Welche Schwierigkeiten oder Hürden sehen Sie, wenn Sie an die Kontaktaufnahme mit einer Stationspsychologin denken?

→ Mitarbeitende MIT ITS-Psychologin:

In welchen Situationen konnte Sie die Psychologin bisher unterstützen?
Woran haben Sie das gemerkt? Wie hat sich das ausgewirkt?
Welche Schwierigkeiten oder Hürden gab es in der Vergangenheit bzgl. Der Kontaktaufnahme mit der Stationspsychologin?

10 Minuten

____
_

Ganz herzlichen Dank, dass Sie sich die Zeit für unser Projekt genommen haben. Bevor wir uns gleich verabschieden:

4: Sind Ihnen im Verlauf des Gesprächs vielleicht Gedanken aufgekommen, die ich noch mit aufnehmen sollte?

Wenn Ihnen im Nachgang noch Dinge wichtig erscheinen oder Sie über unsere heutige Diskussion sprechen möchten, wenden Sie sich an ____________________

3 Minuten

Appendix 3: Interview and Focus Group guides for Substudy 2
Interview guide for former ICU patients

Leitfaden Interview für Patient*innen

Herzlich willkommen und vielen Dank für Ihre Bereitschaft zur Teilnahme.

Vorstellung Moderator*in

Mein Name ist: ______________________________

Gemeinsam mit der Universität Ulm, der Universität Magdeburg und der Charité Berlin führen wir eine Studie in dem Forschungsprojekt „IPS-Pilot: Integrierte psychosoziale Unterstützung in der Intensivmedizin“ durch. Zu diesem Thema möchte ich Ihnen heute einige Fragen stellen, bei denen es um Ihre Erfahrungen und Einschätzungen geht.

Im Rahmen der COVID-19-Pandemie sind auf einigen Intensivstationen in Deutschland Psycholog*innen in die intensivmedizinischen Teams integriert worden, um auf den Stationen mitzuarbeiten. Ihre Aufgabe ist es, Mitarbeitenden Gespräche und andere Interventionen anzubieten, sowie Patient*innen und Angehörige während der intensivmedizinischen Behandlung psychologisch zu betreuen. Darauf möchten wir aufbauen und ein Konzept entwickeln, welches langfristig die psychosoziale Versorgung auf Intensivstationen betrifft. Das heutige Interview findet statt, damit wir von Ihren Erfahrungen lernen können.

Dafür sind 30 bis maximal 60 Minuten eingeplant. Ich werde Sie nun über die Studie und die Datenschutzbestimmungen aufklären.

- Spätere Verwendung der Daten
- Angebot, die Ergebnisse auf Aufforderung an ips.pilot@uniklinik-ulm.de zu schicken
- Form der Dokumentation (Tonband, Transkription, Pseudonymisierung)

Das Tonband ist jetzt eingeschaltet. Würden Sie mir bitte nochmals bestätigten, dass Sie mit der Aufzeichnung des Gesprächs auf Tonband einverstanden sind, dass ich Sie über den Datenschutz aufgeklärt habe und dass alle Angaben freiwillig sind?
Unser Interview besteht aus 4 Leitfragen, zu denen wir manchmal mit vertiefenden Fragen etwas ausführlicher in die Thematik einsteigen werden. Im ersten Teil des Gesprächs wird es um mögliche Belastungen während Ihrer Zeit auf der Intensivstation gehen und in der zweiten Hälfte um Ihren Umgang mit denselben.

Wir beginnen das Interview jetzt.

Appendix 3: Interview and Focus Group guides for Substudy 2

Interview guide for former ICU patients

Aktuelle Situation und aktuelle Probleme (Anforderungen, Ressourcenverlust)

Die erste Frage stützt sich auf die Beobachtung, dass die intensivmedizinische Behandlung von den meisten Patientinnen und Patienten als enorme Belastung erlebt wird.

1: Was genau haben Sie während Ihres Aufenthaltes auf der Intensivstation als besonders belastend erlebt?

Können Sie Beispiele nennen? (Schmerzen, Angst, Hilflosigkeit..)

Wie hat sich die Belastung auf Sie und Ihre Stimmung ausgewirkt?

10 Minuten

Aktuelle Ressourcen (Ressourcen, Organisation)

2. Was hat Ihnen dabei geholfen, mit den Belastungen umzugehen?

z.B.: Besuch, Seelsorge, andere psychosoziale Angebote

5 Minuten

---

3. Standen während Ihres Aufenthaltes auf der Intensivstation mit einer Psychologin in Kontakt?

→ Ja: Wie häufig? Regelmäßig?

Kostete es Sie Überwindung, mit der Psychologin in Kontakt zu treten?

- Wenn ja: Warum?
- Wie kam der Kontakt zustande?

3.1 Was hat die Psychologin beeinflusst/verändert/bewirkt?

→ Falls Sie den Kontakt als unterstützend erlebt haben, was genau hat Ihnen gutgetan?

→ Falls Sie den Kontakt (auch mal) als weniger unterstützend erlebt haben, was genau hat Ihnen gefehlt?

3.2 In welchen Situationen/Wegen welcher Anliegen haben Sie mit der Psychologin gesprochen?

z.B. bei Aufnahme/Entlassung, Begleitung von Besuchen, präoperativ, bei ärztl. Gesprächen

→ Wie genau konnte die Psychologin Sie in Ihrem Anliegen unterstützen?

→ Wie hat sich das auf Sie ausgewirkt?

→ Woran haben Sie das gemerkt?

3.3 Gab es Schwierigkeiten oder Hürden im Kontakt mit der Psychologin?

- Wenn ja: Welche?

z.B. Verfügbarkeit, Privatsphäre

→ Nein: Wenn Sie auf die Zeit auf der Intensivstation zurückblicken, würden Sie sagen, dass Sie von einer psych. Begleitung hätten profitieren können?

- Wenn ja: In welchen Situationen hätte Ihnen die Psychologin eine Unterstützung sein können?
- Was hätte eine Psychologin für Sie tun können?
- Wie hätte sich das womöglich auf Sie ausgewirkt?
- Woran hätten Sie das gemerkt?

Appendix 3: Interview and Focus Group guides for Substudy 2
Interview guide for former ICU patients

15 Minuten

____

Ganz herzlichen Dank, dass Sie sich die Zeit für unser Projekt genommen haben. Bevor wir uns gleich verabschieden:

4: Sind Ihnen im Verlauf des Gesprächs vielleicht Gedanken aufgekommen, die ich noch mit aufnehmen sollte?

Wenn Ihnen im Nachgang noch Dinge wichtig erscheinen oder Sie über unsere heutige Diskussion sprechen möchten, wenden Sie sich an ____________________

3 Minuten

Appendix 3: Interview and Focus Group guides for Substudy 2
Interview guide for relatives of former ICU patients

Leitfaden Interview für Angehörige

Herzlich willkommen und vielen Dank für Ihre Bereitschaft zur Teilnahme.

Vorstellung Moderator*in

Mein Name ist: ______________________________

Gemeinsam mit der Universität Ulm, der Universität Magdeburg und der Charité Berlin führen wir eine Studie in dem Forschungsprojekt „IPS-Pilot: Integrierte psychosoziale Unterstützung in der Intensivmedizin“ durch. Zu diesem Thema möchte ich Ihnen heute einige Fragen stellen, bei denen es um Ihre Erfahrungen und Einschätzungen geht.

Im Rahmen der COVID-19-Pandemie sind auf einigen Intensivstationen in Deutschland Psycholog*innen in die intensivmedizinischen Teams integriert worden, um auf den Stationen mitzuarbeiten. Ihre Aufgabe ist es, Mitarbeitenden Gespräche und andere Interventionen anzubieten, sowie Patient*innen und Angehörige während der intensivmedizinischen Behandlung psychologisch zu betreuen. Darauf möchten wir aufbauen und ein Konzept entwickeln, welches langfristig die psychosoziale Versorgung auf Intensivstationen betrifft. Das heutige Interview findet statt, damit wir von Ihren Erfahrungen lernen können.

Dafür sind 30 bis maximal 60 Minuten eingeplant. Ich werde Sie nun über die Studie und die Datenschutzbestimmungen aufklären.

- Spätere Verwendung der Daten
- Angebot, die Ergebnisse auf Aufforderung an ips.pilot@uniklinik-ulm.de zu schicken
- Form der Dokumentation (Tonband, Transkription, Pseudonymisierung)

Das Tonband ist jetzt eingeschaltet. Würden Sie mir bitte nochmals bestätigten, dass Sie mit der Aufzeichnung des Gesprächs auf Tonband einverstanden sind, dass ich Sie über den Datenschutz aufgeklärt habe und dass alle Angaben freiwillig sind?
Unser Interview besteht aus 4 Leitfragen, zu denen wir manchmal mit vertiefenden Fragen etwas ausführlicher in die Thematik einsteigen werden. Im ersten Teil des Gesprächs wird es um mögliche Belastungen während der intensivmedizinischen Behandlung Ihres/r Angehörigen gehen und in der zweiten Hälfte um Ihren Umgang mit denselben.

Wir beginnen das Interview jetzt.

Appendix 3: Interview and Focus Group guides for Substudy 2

Interview guide for relatives of former ICU patients

Aktuelle Situation und aktuelle Probleme (Anforderungen, Ressourcenverlust)

Die erste Frage stützt sich auf die Beobachtung, dass die intensivmedizinische Behandlung nicht nur von den meisten Patient*innen, sondern auch von vielen Angehörigen als ungeheure Belastung erlebt wird.

1: Was genau haben Sie während des Aufenthaltes der Ihnen nahestehenden Person auf der Intensivstation als belastend erlebt?

Können Sie Beispiele nennen? (Angst, Hilflosigkeit, Überforderung..)

Wie hat sich die Belastung auf Sie, Ihre Stimmung und Ihren Alltag ausgewirkt?

10 Minuten

Aktuelle Ressourcen (Ressourcen, Organisation)

2. Was hat Ihnen dabei geholfen, mit den Belastungen umzugehen?

z.B.: Austausch mit nahestehenden Personen, Seelsorge, andere psychosoziale Angebote

5 Minuten

---

3. Standen Sie während der intensivmedizinischen Behandlung Ihres/r Angehörigen mit einer Psychologin in Kontakt?

→ Ja: Wie häufig? Regelmäßig?

Kostete es Sie Überwindung, mit der Psychologin in Kontakt zu treten?

- Wenn ja: Warum?
- Wie kam der Kontakt zustande?

3.1 Was hat die Psychologin beeinflusst/verändert/bewirkt?

→ Falls Sie den Kontakt als unterstützend erlebt haben, was genau hat Ihnen gutgetan?

→ Falls Sie den Kontakt (auch mal) als weniger unterstützend erlebt haben, was genau hat Ihnen gefehlt?

3.2 In welchen Situationen/Wegen welcher Anliegen haben Sie mit der Psychologin gesprochen?

z.B. in besonders belastenden Situationen, bei der Begleitung von Besuchen, bei ärztl. Gesprächen

→ Wie genau konnte die Psychologin Sie unterstützen?

→ Wie hat sich das auf Sie ausgewirkt?

→ Woran haben Sie das gemerkt?

3.3 Gab es Schwierigkeiten oder Hürden im Kontakt mit der Psychologin?

- Wenn ja: Welche?

z.B. Verfügbarkeit, Privatsphäre

→ Nein: Wenn Sie auf die Zeit der intensivmedizinischen Behandlung ihres/r Angehörigen zurückblicken, würden Sie sagen, dass Sie von einer psych. Begleitung hätten profitieren können?

- Wenn ja: In welchen Situationen hätte Ihnen die Psychologin eine Unterstützung sein können?
- Was hätte eine Psychologin für Sie tun können?
- Wie hätte sich das womöglich auf Sie ausgewirkt?
- Woran hätten Sie das gemerkt?

15 Minuten

Ganz herzlichen Dank, dass Sie sich die Zeit für unser Projekt genommen haben. Bevor wir uns gleich verabschieden:

4: Sind Ihnen im Verlauf des Gesprächs vielleicht Gedanken aufgekommen, die ich noch mit aufnehmen sollte?

Wenn Ihnen im Nachgang noch Dinge wichtig erscheinen oder Sie über unsere heutige Diskussion sprechen möchten, wenden Sie sich an ____________________

3 Minuten

Appendix 3: Interview and Focus Group guides for Substudy 2

Focus Group guide for former ward psychologists

Phase		Fragestellungen	Zeit/V
Einleitung	0	- Begrüßung<br>- Fragen, ob kollegiales Du für alle in Ordnung<br>- Vorstellung Moderator*innen und Rollenverteilung (GK: Moderator, SK: Co-Moderator, LN: Notiert Sprechreihenfolge)<br><br>- Vorstellung Projekt (Universität Ulm, Universität Magdeburg und Charité Berlin)<br>- Im Rahmen der COVID-19-Pandemie sind auf einigen Intensivstationen in Deutschland Psycholog*innen in die intensivmedizinischen Teams integriert worden, um auf den Stationen mitzuarbeiten. Studien zeigen, dass die emotionale und körperliche Belastung von Patient:innen, Angehörigen und Mitarbeitenden dort hoch sein kann. Deshalb möchten wir ein Konzept entwickeln, Mitarbeitende, Patient:innen und Angehörigen dort zu unterstützen.<br><br>- Ihr seid eingeladen worden, weil ihr Psycholog:innen seid, die schon Erfahrung mit der Arbeit auf Intensivstationen haben. Wir möchten von euren Erfahrungen und eurem Wissen lernen.<br><br>Ich möchte in unserer heutigen Gruppendiskussion gern einige Themen rund um Belastungen auf ITS und Möglichkeiten, wie man ihnen begegnen kann, besprechen. Dafür sind etwa 90 Minuten eingeplant.<br><br>- Wir freuen uns über eure Einblicke, teilt gern eure Erfahrungen und Gedanken oder nehmt gern Bezug auf das, was in der Gruppe gesagt wird<br>- Wir wollen versuchen, ob es ohne tatsächliches oder „virtuelles“ Melden klappt. Sprecht einfach frei los.	5 Min.

Appendix 3: Interview and Focus Group guides for Substudy 2

Focus Group guide for former ward psychologists

Aufklärung	0	- Tonbandaufnahme + Aufnahme über Zoom (separat Video und Audio, Video wird direkt gelöscht)<br>- Wir werden in unseren Berichten keine Namen nennen und das, was ihr sagt, vertraulich behandeln.<br>- Die Tonaufnahme wird transkribiert und pseudonymisiert ausgewertet.<br>- Die Daten werden später verwendet, um das Konzept für die integrierte Versorgung auszuarbeiten<br>- Infos in Einwilligungserklärungen, dazu Fragen?<br><br>**ZETTEL & STIFT FÜR SPRECHERREIHENFOLGE BEREITHALTEN**	
Vorstellung	0	Gut, fangen wir an.<br>Die Tonaufnahme startet jetzt. **TONBAND EINSCHALTEN**<br><br>- Vorstellung der Reihe nach<br>- Name<br>- Funktion<br>- Klinik<br>- Fachabteilung<br>- Arbeitserfahrung als feste/r Psycholog*in im Hochintensitätsbereich (kurz)<br>- Namen in Kachel eingeben, damit alle füreinander ansprechbar sind	
Eröffnungsfrage: Erlebte Belastungen	1	**Ihr wart oder seid eingestellt, um Mitarbeitende, Patient*innen und Angehörige im Umgang mit ihren Belastungen im ITS-Kontext zu unterstützen. Wir möchten gerne wissen, welche Belastungen ihr dabei wahrnehmt.**<br><br>**Was wird aus eurer Sicht von Mitarbeitenden als belastend erlebt?**<br><br>**Was wird aus eurer Sicht von Patient*innen als belastend erlebt?**<br><br>**Was wird aus eurer Sicht von Angehörigen als belastend erlebt?**	10

Appendix 3: Interview and Focus Group guides for Substudy 2

Focus Group guide for former ward psychologists

Übergangsfrage: Chancen	2	**Denkt bitte an eure Erfahrungen mit der Tätigkeit als Stationspsychologin oder Stationspsychologe. Welche Chancen seht ihr darin, dass Stationspsychologen für den Umgang mit den Belastungen auf der ITS ins Team integriert werden?**<br><br>- alle drei Gruppen besprochen?<br><br>- Welche Möglichkeiten gibt es, mit den Belastungen auf der ITS umzugehen?<br>- Was waren Möglichkeiten/ was hat gut geklappt/ was war erfolgreich?<br>- Integration ins Team<br>- Liegt eine Chance darin, dass die Stationspsychologin fest ins Team integriert ist? Was macht die Rolle der Psychologin im Team aus?<br>- Im Vergleich zu anderen Versorgungsformen…?<br>- Gibt es Vorteile einer psychosozialen Versorgung durch eine fest ins Team integrierte Psychologin im Gegensatz zu anderen Versorgungsformen? z.B. Liaison-Dienst, Konsildienst, Seelsorge, Peer-Support?<br>-<br><br>- therapeutischen Inhalte? Wie kann den Betroffenen geholfen werden?<br><br>- Hast Du vielleicht ein Beispiel?<br>- Was denken die anderen?<br>- Kannst du das näher ausführen? / Kannst du mir sagen, was du mit … meinst?	20 min

Appendix 3: Interview and Focus Group guides for Substudy 2

Focus Group guide for former ward psychologists

Überleitungsfrage 2: Herausforderungen		**Welche Herausforderungen gibt es in der Arbeit als Stationspsycholog*in bei der Unterstützung von Mitarbeitenden, Patient*innen und Angehörigen?**<br>- Alle drei Gruppen besprochen?<br>- strukturell (Arbeitszeiten, räumliche Situation…)<br>- Was sind Grenzen?<br>- Behandlungsinhalte?<br>- Welche psychologischen Methoden sind herausfordernder anzuwenden?<br>- Erlebt ihr auch selbst Belastungen durch die Arbeit auf der ITS?<br><br>○ Was denken die anderen?<br>○ Kannst du das näher ausführen? / Kannst du mir sagen, was du mit … meinst?<br>○ Gibt es Beispiele?	20 min
Hauptfrage: Verbesserungs-möglichkeiten und Interventions-entwicklung	3	**Ziel des IPS-Pilot-Projekts ist es, eine Intervention zu entwickeln, die eine möglichst ideale psychosoziale Versorgung auf ITS gewährleisten soll. Wie würde für euch die ideale psychosoziale Versorgung auf der ITS aussehen?**<br><br>- Bezug nehmen auf o.g. Herausforderungen: Ihr habt gesagt, dass XY in der Arbeit mit XY schwierig war - wie könnte man das lösen, was würdet ihr brauchen, damit ihr gut damit umgehen könnt?<br>- Was könnte die Rolle der Stationspsychologin sein?<br>- Was könnten Aufgaben der Stationspsychologin sein?<br><br>○ Was denken die anderen?<br>○ Kannst du das näher ausführen? / Kannst du mir sagen, was du mit … meinst?<br>○ Gibt es Beispiele?	20 Min.

Appendix 3: Interview and Focus Group guides for Substudy 2

Focus Group guide for former ward psychologists

Hauptfrage 2: Ergänzende Maßnahmen	4	**Wären eurer Meinung nach neben einer Stationspsychologin noch weitere Maßnahmen notwendig, um die psychosoziale Versorgung zu gewährleisten?**<br><br>- Habt ihr Erfahrungen in der Zusammenarbeit mit anderen psychosozialen Interventionen?<br>- Wo sind Schnittstellen und Unterschiede?<br>- (Wobei) habt ihr *X psychosoziale Intervention X* als hilfreich empfunden?<br>- Was sind auch hier in der Zusammenarbeit Schwierigkeiten und Chancen?	10 Min.
Ende	5	**Wir nähern uns dem Ende. Kommt noch etwas hinzu, das noch berücksichtigt werden sollte?**<br><br>**Okay, kommen wir zum Abschluss: Von all den Dingen, die wir besprochen haben, was ist für Euch das Wichtigste?**<br><br>**Möchte noch jemand ein abschließendes Wort sagen?**<br><br>- Bedankung<br>- Nochmal zur Erinnerung: Wenn ihr Interesse am Verlauf der Studie habt, könnt ihr eine E-Mail an ips.pilot@uniklinik-ulm.de schreiben<br><br>Tonaufnahme stoppen	5 Min.

Appendix 3: Interview and Focus Group guides for Substudy 2

Focus Group guide for HCPs with experience in working with a ward psychologist

Phase		Fragestellungen	Zeit/V
Einleitung	0	- Begrüßung<br>- Fragen, ob kollegiales Du für alle in Ordnung (wurde bereits in Mail erwähnt)<br>- Vorstellung Moderator*innen und Rollenverteilung (GK: Moderator, SK: Co-Moderator, ggf. medizinische Doktorandin: Notiert Sprechreihenfolge)<br><br>- Vorstellung Projekt (Universität Ulm, Universität Magdeburg und Charité Berlin)<br><br>- Studien zeigen, dass die emotionale und körperliche Belastung von Patient*innen, Angehörigen und Mitarbeitenden auf Intensivstationen hoch sein kann. Um mit diesen Belastungen umzugehen, sind im Rahmen der COVID-19-Pandemie auf einigen Intensivstationen in Deutschland bereits Psycholog*innen in die intensivmedizinischen Teams integriert worden.<br>- Im IPS-Pilot Projekt möchten wir nun überprüfen, ob diese Art der Versorgung auf Intensivstationen generell hilfreich und machbar wäre.<br><br>- Ihr seid eingeladen worden, weil ihr als Mitarbeitende auf Intensivstationen bereits Erfahrungen mit einer Stationspsychologin gemacht habt. Ich möchte in unserer heutigen Gruppendiskussion gern einige Themen rund um Belastungen auf ITS und Möglichkeiten, wie man ihnen begegnen kann, besprechen. Dafür sind etwa 90 Minuten eingeplant.<br><br>- Wir freuen uns über eure Einblicke, teilt gern eure Erfahrungen und Gedanken oder nehmt gern Bezug auf das, was in der Gruppe gesagt wird<br>- Wir wollen versuchen, ob es ohne tatsächliches oder „virtuelles“ Melden klappt. Sprecht einfach frei los.	5 Min.

Appendix 3: Interview and Focus Group guides for Substudy 2

Focus Group guide for HCPs with experience in working with a ward psychologist

Aufklärung	0	- Tonbandaufnahme + Aufnahme über Zoom (separat Video und Audio, Video wird direkt gelöscht)<br>- Wir werden in unseren Berichten keine Namen nennen und das, was ihr sagt, vertraulich behandeln.<br>- Die Tonaufnahme wird transkribiert und pseudonymisiert ausgewertet.<br>- Die Daten werden später verwendet, um das Konzept für die integrierte Versorgung auszuarbeiten<br>- Infos in Einwilligungserklärungen, dazu Fragen?<br><br>**ZETTEL & STIFT FÜR SPRECHERREIHENFOLGE BEREITHALTEN**	
Vorstellung	0	Gut, fangen wir an.<br>Die Tonaufnahme startet jetzt. **TONBAND EINSCHALTEN**<br><br>- Vorstellung der Reihe nach<br>- Name<br>- Funktion<br>- Klinik<br>- Fachabteilung<br>- Arbeitserfahrung als Mitarbeiter*in im Hochintensitätsbereich (kurz)	
Eröffnungsfrage: Erlebte Belastungen	1	**Ihr seid Mitarbeitende im intensivmedizinischen Kontext. Wir möchten gerne wissen, was dabei für euch belastend ist.**<br>- Was daran ist besonders belastend<br>- Habt ihr Beispiele?<br>- Kannst du das ausführen?<br>- Wie erleben das die anderen?	10

Appendix 3: Interview and Focus Group guides for Substudy 2

Focus Group guide for HCPs with experience in working with a ward psychologist

Übergangsfrage: Erfahrung mit Stat.Psych.	2	**Denkt bitte an eure Erfahrungen mit der Stationspsychologin oder dem Stationspsychologen. Welche Erfahrungen habt ihr gemacht?**<br><br>- Was habt ihr daran als hilfreich empfunden?<br>- Was war herausfordernd?<br>- Was hat sich durch die Stationspsychologin geändert?/Wie hat es sich angefühlt?<br>- Was waren Möglichkeiten/ was hat gut geklappt/ was war erfolgreich?<br><br>- Hast Du vielleicht ein Beispiel?<br>- Was denken die anderen?<br>- Kannst Du das näher ausführen? / Kannst Du mir sagen, was Du mit … meinst?	20 min

Appendix 3: Interview and Focus Group guides for Substudy 2

Focus Group guide for HCPs with experience in working with a ward psychologist

Hauptfrage: Interventionsentwicklung		**Ziel des IPS-Pilot-Projekts ist es, eine Intervention zu entwickeln, die eine möglichst ideale psychosoziale Versorgung auf ITS gewährleisten soll. Wenn es keine Grenzen gäbe, wie würde für euch die ideale psychosoziale Versorgung auf der ITS aussehen?**<br>- Ihr habt gesagt, es gibt z.B. folgende Belastungen: XY - wie könnte man das lösen, was würdet ihr brauchen, damit ihr gut damit umgehen könnt?<br>- Wie könnte die Stationspsychologin das Team stärken, um besser mit diesen Belastungen umzugehen?<br>- Was könnten dabei Aufgaben der Stationspsychologin sein?<br>- Wie würde eine Arbeitsplatzbeschreibung für sie aussehen?/Wie wäre die Stelle konkret organisiert? (z.B. Arbeitszeit, Anwesenheit, Erreichbarkeit)?<br>- Welche strukturellen Maßnahmen oder Angebote sollten dazugehören (regelmäßige Supervisionen, Hotline, Gesundheitszirkel, Peer-Support-System)<br>○ Was denken die anderen?<br>○ Kannst du das näher ausführen?/Kannst du mir sagen, was du mit … meinst?<br>○ Gibt es Beispiele?<br>○ Wofür wäre XY hilfreich/Was wäre dadurch anders?<br>○ Wie sollte XY genau aussehen?	20 min
Hauptfrage 2: Machbarkeit	3	**Wenn wir nun versuchen würden, die Ideen der idealen Intervention in die reale Welt zu überführen – was könnte daran nicht funktionieren?**<br>- Welche Schwierigkeiten könnten dabei auf uns warten?<br>- Gibt es Möglichkeiten, wie es doch funktionieren könnte?<br>- Was denken die anderen?<br>- Kannst du das näher ausführen? / Kannst du mir sagen, was du mit … meinst?<br>- Gibt es Beispiele?	20 Min.

Appendix 3: Interview and Focus Group guides for Substudy 2

Focus Group guide for HCPs with experience in working with a ward psychologist

Ende	4	**Wir nähern uns dem Ende. Kommt noch etwas hinzu, das noch berücksichtigt werden sollte?**<br><br>**Okay, kommen wir zum Abschluss: Von all den Dingen, die wir besprochen haben, was ist für Euch das Wichtigste?**<br><br>**Möchte noch jemand ein abschließendes Wort sagen?**<br><br>- Bedankung<br>- Nochmal zur Erinnerung: Wenn ihr Interesse am Verlauf der Studie habt, könnt ihr eine E-Mail an ips.pilot@uniklinik-ulm.de schreiben<br><br>Tonaufnahme stoppen	5 Min.

Appendix 3: Interview and Focus Group guides for Substudy 2
Focus Group guide for HCPs without experience in working with a ward psychologist

Phase		Fragestellungen	Zeit/V
Einleitung	0	- Begrüßung<br>- Fragen, ob kollegiales Du für alle in Ordnung (wie in Mail erwähnt)<br>- Vorstellung Moderator*innen und Rollenverteilung (GK: Moderator, SK: Co-Moderator, ggf. medizinische Doktorandin: Notiert Sprechreihenfolge)<br><br>- Vorstellung Projekt (Universität Ulm, Universität Magdeburg und Charité Berlin)<br><br>- Studien zeigen, dass die emotionale und körperliche Belastung von Patient*innen, Angehörigen und Mitarbeitenden auf Intensivstationen hoch sein kann. Um mit diesen Belastungen umzugehen, sind im Rahmen der COVID-19-Pandemie auf einigen Intensivstationen in Deutschland bereits Psycholog*innen in die intensivmedizinischen Teams integriert worden.<br>- Im IPS-Pilot Projekt möchten wir nun überprüfen, ob diese Art der Versorgung auf Intensivstationen generell hilfreich und machbar wäre.<br><br>- Ihr seid eingeladen worden, weil ihr auf Intensivstationen mitarbeitet. Wir möchten gerne von den Erfahrungen, die ihr dort macht, lernen. Ich möchte in unserer heutigen Gruppendiskussion gern einige Themen rund um Belastungen auf ITS und Möglichkeiten, wie man ihnen begegnen kann, besprechen. Dafür sind etwa 90 Minuten eingeplant.<br><br>- Wir freuen uns über eure Einblicke, teilt gern eure Erfahrungen und Gedanken oder nehmt gern Bezug auf das, was in der Gruppe gesagt wird<br>- Wir wollen versuchen, ob es ohne tatsächliches oder „virtuelles“ Melden klappt. Sprecht einfach frei los.	5 Min.

Appendix 3: Interview and Focus Group guides for Substudy 2

Focus Group guide for HCPs without experience in working with a ward psychologist

Aufklärung	0	- Tonbandaufnahme + Aufnahme über Zoom (separat Video und Audio, Video wird direkt gelöscht)<br>- Wir werden in unseren Berichten keine Namen nennen und das, was ihr sagt, vertraulich behandeln.<br>- Die Tonaufnahme wird transkribiert und pseudonymisiert ausgewertet.<br>- Die Daten werden später verwendet, um das Konzept für die integrierte Versorgung auszuarbeiten<br>- Infos in Einwilligungserklärungen, dazu Fragen?<br><br>**ZETTEL & STIFT FÜR SPRECHERREIHENFOLGE BEREITHALTEN**	5 Min.
Vorstellung	0	Gut, fangen wir an.<br>Die Tonaufnahme startet jetzt. **TONBAND EINSCHALTEN**<br><br>- Vorstellung der Reihe nach<br>- Name<br>- Funktion<br>- Klinik<br>- Fachabteilung<br>- Arbeitserfahrung als Mitarbeiter*in im Hochintensitätsbereich (kurz)	5 Min.
Eröffnungsfrage: Erlebte Belastungen	1	**Ihr seid Mitarbeitende im intensivmedizinischen Kontext. Wir möchten gerne wissen, was dabei für euch belastend ist.**<br><br>- Was daran ist besonders belastend?<br>- Habt ihr Beispiele?<br>- Kannst du das ausführen?<br>- Wie erleben das die anderen?	10-15 Min.

Appendix 3: Interview and Focus Group guides for Substudy 2

Focus Group guide for HCPs without experience in working with a ward psychologist

Hauptfrage: Interventionsentwicklung	2	**Ziel des IPS-Pilot-Projekts ist es, eine Intervention zu entwickeln, die eine möglichst ideale psychosoziale Versorgung auf ITS gewährleisten soll. Wenn es keine Grenzen gäbe, wie würde für euch die ideale psychosoziale Versorgung auf der ITS aussehen?**<br>- Ihr habt gesagt, es gibt z.B. folgende Belastungen: XY - wie könnte man das lösen, was würdet ihr brauchen, damit ihr gut damit umgehen könnt?<br>- Wie könnte die Stationspsychologin das Team stärken, um besser mit diesen Belastungen umzugehen?<br>- Was könnten dabei Aufgaben der Stationspsychologin sein?<br>- Wie würde eine Arbeitsplatzbeschreibung für sie aussehen?/Wie wäre die Stelle konkret organisiert? (z.B. Arbeitszeit, Anwesenheit, Erreichbarkeit)?<br>- Welche strukturellen Maßnahmen oder Angebote sollten dazugehören (regelmäßige Supervisionen, Hotline, Gesundheitszirkel, Peer-Support-System)<br><br>**(Falls Möglichkeit einer Stationspsychologin nicht spontan genannt, stellen:) Auf einigen ITS in Deutschland gibt es fest ins Team integrierte Stationspsycholog*innen, um Mitarbeitende zu stärken und Patient*innen und Angehörige zu entlasten. Was denkt ihr darüber?**<br><br>○ Was denken die anderen?<br>○ Kannst du das näher ausführen?/Kannst du mir sagen, was du mit … meinst?<br>○ Gibt es Beispiele?<br>○ Wofür wäre XY hilfreich/Was wäre dadurch anders?<br>○ Wie sollte XY genau aussehen?<br>○ Gibt es noch andere Ideen, die Ressourcen der betroffenen Personen zu stärken?	30 min
Hauptfrage 2: Machbarkeit	3	**[Evtl. die Ideen aus Frage 2 zusammenfassen. Sie haben gesagt, dass…]. Wenn wir nun versuchen würden, die Ideen der idealen Intervention in die reale Welt zu überführen – was könnte daran nicht funktionieren?**<br>- Welche Schwierigkeiten könnten dabei auf uns warten?<br>- Gibt es Möglichkeiten, wie es doch funktionieren könnte?<br><br>- Was denken die anderen?<br>- Kannst du das näher ausführen? / Kannst du mir sagen, was du mit … meinst?	25-30 Min.

Appendix 3: Interview and Focus Group guides for Substudy 2
Focus Group guide for HCPs without experience in working with a ward psychologist

		- Gibt es Beispiele?<br>-	
Ende	4	**Wir nähern uns dem Ende. Kommt noch etwas hinzu, das noch berücksichtigt werden sollte?**<br><br>**Okay, kommen wir zum Abschluss: Von all den Dingen, die wir besprochen haben, was ist für Euch das Wichtigste?**<br><br>**Möchte noch jemand ein abschließendes Wort sagen?**<br><br>- Bedankung<br>- Nochmal zur Erinnerung: Wenn ihr Interesse am Verlauf der Studie habt, könnt ihr eine E-Mail an ips.pilot@uniklinik-ulm.de schreiben<br><br>Tonaufnahme stoppen	5 Min.

Appendix 3: Interview and Focus Group guides for Substudy 2
Focus Group guide for former ICU patients and relatives

Phase		Fragestellungen	Zeit/V
Einleitung	0	- Begrüßung<br>- Vorstellung Moderator*innen und Rollenverteilung (GK: Moderator, SK: Co-Moderator, ggf. medizinische Doktorandin: Notiert Sprechreihenfolge)<br><br>- Vorstellung Projekt (Universität Ulm, Universität Magdeburg und Charité Berlin)<br><br>- Studien zeigen, dass die emotionale und körperliche Belastung von Patient*innen, Angehörigen und Mitarbeitenden auf Intensivstationen hoch sein kann. Um mit diesen Belastungen umzugehen, sind im Rahmen der COVID-19-Pandemie auf einigen Intensivstationen in Deutschland bereits Psycholog*innen in die intensivmedizinischen Teams integriert worden.<br>- Im IPS-Pilot Projekt möchten wir nun überprüfen, ob diese Art der Versorgung auf Intensivstationen generell hilfreich und machbar wäre.<br><br>- Sie sind eingeladen worden, weil Sie als Patienten oder Angehörige den Kontext der Intensivstation miterlebt haben und dabei teilweise auch schon Erfahrungen mit einer Stationspsychologin gemacht haben. Ich möchte in unserer heutigen Gruppendiskussion gern einige Themen rund um Belastungen auf ITS und Möglichkeiten, wie man ihnen begegnen kann, besprechen. Dafür sind etwa 90 Minuten eingeplant.<br><br>- Wir freuen uns über Ihre Einblicke, teilen Sie gern Ihre Erfahrungen und Gedanken oder nehmt gern Bezug auf das, was in der Gruppe gesagt wird.<br>- Es kann belastend sein, über die Erfahrungen auf der Intensivstation zu sprechen. Wenn Sie gern eine Pause machen möchten, ist das natürlich jederzeit möglich. Ihre Teilnahme ist freiwillig und falls Sie währenddessen die Teilnahme beenden möchten, ist das jederzeit und ohne Konsequenzen möglich.	5 Min.

Appendix 3: Interview and Focus Group guides for Substudy 2

Focus Group guide for former ICU patients and relatives

Aufklärung	0	- Tonbandaufnahme<br>- Wir werden in unseren Berichten keine Namen nennen und das, was Sie sagen, vertraulich behandeln.<br>- Die Tonaufnahme wird transkribiert und pseudonymisiert ausgewertet. → Es werden keine Rückschlüsse auf Ihre Person möglich sein<br>- Die Daten werden später verwendet, um das Konzept für die integrierte Versorgung auszuarbeiten<br>- Infos in Einwilligungserklärungen, dazu Fragen?<br><br>**ZETTEL & STIFT FÜR SPRECHERREIHENFOLGE BEREITHALTEN**	5 min
Vorstellung	0	Gut, fangen wir an.<br>Die Tonaufnahme startet jetzt. **TONBAND EINSCHALTEN**<br><br>- Vorstellung der Reihe nach<br>- Name<br>- Funktion: Patient oder Angehörige<br>- Wenn Sie möchten: Wie lange dauerte die Behandlung und in welcher Klinik waren Sie?	
Eröffnungsfrage: Erlebte Belastungen	1	**Sie haben die Behandlung im intensivmedizinischen Kontext als behandelte oder angehörige Personen miterlebt. Wir möchten gerne wissen, was Sie dabei als belastend empfunden haben.**<br><br>- Was daran ist besonders belastend?<br>- Haben Sie dazu ein Beispiel?<br>- Möchten Sie das näher ausführen?<br>- Wie erleben das die anderen?	15-20

Appendix 3: Interview and Focus Group guides for Substudy 2

Focus Group guide for former ICU patients and relatives

Übergangsfrage: Erfahrung mit Stat.Psych.	2	**Haben Sie Erfahrungen mit Unterstützungsangeboten der Klinik, um mit diesen Belastungen umzugehen, gemacht?**<br><br>**Einige von Ihnen hatten während dieser Zeit Kontakt zu einer im Intensivteam integrierten Stationspsychologin. Wie haben Sie das empfunden?**<br><br>- Was haben Sie daran als hilfreich empfunden?<br>- Was war herausfordernd?<br>- Hat sich durch das Angebot / die Stationspsychologin etwas geändert?<br><br>- Haben Sie vielleicht ein Beispiel?<br>- Was denken die anderen?<br>- Möchten Sie das näher ausführen? / Können Sie mir sagen, was Sie mit … meinen?	20 min

Appendix 3: Interview and Focus Group guides for Substudy 2

Focus Group guide for former ICU patients and relatives

Hauptfrage: Interventionsentwicklung		**Ziel des IPS-Pilot-Projekts ist es, eine Intervention zu entwickeln, die eine möglichst ideale psychosoziale Unterstützung auf ITS gewährleisten soll. Wenn es keine Grenzen gäbe, wie würde für Sie diese ideale Versorgung auf der ITS aussehen?**<br>- Sie haben gesagt, es gibt z.B. folgende Belastungen: XY - wie könnte man das lösen, was hätten Sie als Patienten und Angehörige gebraucht, damit Sie gut damit umgehen können?<br>- Gibt es noch weitere Angebote, die notwendig wären? (z.B. Nachsorge, Vermittlung an externe Angebote, …)<br>- Wie könnte die Stationspsychologin Patienten oder Angehörigen helfen, um besser mit diesen Belastungen umzugehen?<br>- Welche Rahmenbedingungen bräuchte es für die Stationspsychologin? (z.B. Arbeitszeit, Anwesenheit, Erreichbarkeit)?<br>o Was denken die anderen?<br>o Möchten Sie das näher ausführen?/ Können Sie mir sagen, was Sie mit … meinen?<br>o Gibt es Beispiele?<br>o Wofür wäre XY hilfreich/Was wäre dadurch anders?<br>o Wie sollte XY genau aussehen?	20 min
Hauptfrage 2: Machbarkeit	3	**Wenn wir nun versuchen würden, die Ideen der idealen Intervention in die reale Welt zu überführen – was könnte daran nicht funktionieren?**<br>- Gibt es Situationen oder Umstände, in denen es schwer fallen könnte, die Unterstützung zu nutzen?<br>- Welche Schwierigkeiten könnten dabei auf uns warten?<br>- Gibt es Möglichkeiten, wie es doch funktionieren könnte?<br>- Was denken die anderen?<br>- Möchten Sie das näher ausführen? / Können Sie mir sagen, was Sie mit … meinen?<br>- Gibt es Beispiele?	15-20 Min.

Appendix 3: Interview and Focus Group guides for Substudy 2
Focus Group guide for former ICU patients and relatives

Ende	4	**Wir nähern uns dem Ende. Kommt noch etwas hinzu, das noch berücksichtigt werden sollte?**<br><br>**Okay, kommen wir zum Abschluss: Von all den Dingen, die wir besprochen haben, was ist für Sie das Wichtigste?**<br><br>**Möchte noch jemand ein abschließendes Wort sagen?**<br><br>- Bedankung<br>- Nochmal zur Erinnerung: Wenn Sie Interesse am Verlauf der Studie haben, können Sie eine E-Mail an ips.pilot@uniklinik-ulm.de schreiben<br><br>- Wenn Sie im Nachgang an die Gruppe Gesprächsbedarf haben, können Sie sich an katrin.schuermann@charite.de wenden<br><br>Tonaufnahme stoppen	5 Min.

Appendix 3: Interview and Focus Group guides for Substudy 2
Focus Group guide for participants with expertise in clinical structures

Phase		Fragestellungen	Zeit/V
Einleitung	0	- Begrüßung<br>- Vorstellung Moderator*innen und Rollenverteilung (SK/GK/HH; Moderation und Co-Moderation) Ggf. medizinische Doktorandin: Notiert Sprechreihenfolge)<br><br>- Vorstellung Projekt (Universität Ulm, Universität Magdeburg und Charité Berlin)<br>- Studien zeigen, dass die emotionale und körperliche Belastung von Patient*innen, Angehörigen und Mitarbeitenden auf Intensivstationen hoch sein kann. Dabei konnte gezeigt werden, dass diese Belastungen bei Mitarbeitenden Einfluss auf die Arbeitsqualität, die Anzahl der Krankentage sowie sogar Wechsel in andere Berufsfelder haben. Auf Patient*innenseite führt Belastung im Zusammenhang mit intensivmedizinischer Behandlung nachweislich u.a. zu psychischen Folgeerscheinungen und damit zur Notwendigkeit von Anschlussbehandlung.<br>- Während der COVID-19 Pandemie waren diese Belastungen um ein vielfaches gesteigert, sodass während dieser Zeit auf einigen Intensivstationen in Deutschland Psycholog*innen in die intensivmedizinischen Teams integriert wurden.<br>- Das IPS-Pilot Projekt ist eine Machbarkeitsstudie, das heißt, wir möchten überprüfen, ob diese Art der Versorgung generell auf Intensivstationen hilfreich und umsetzbar wäre.<br><br>- Sie sind eingeladen worden, weil Sie spezifische strukturelle Expertise in der klinischen Versorgung haben. Wir möchten über Ihre Einstellungen, Ihre Erfahrungen und Ihr Wissen sprechen und davon lernen. Wir würden gern Ihre Einschätzung aus Ihren verschiedenen – teilweise auch mehreren – Rollen, die Sie in Ihrer Klinik einnehmen, hören.<br>- Wir sind Ihnen daher sehr dankbar, dass Sie sich Zeit für dieses wichtige Thema nehmen und uns so bei der Verbesserung der psychosozialen Versorgung helfen. Für unsere Gruppendiskussion sind etwa 90 Minuten eingeplant.<br><br>- Wir freuen uns über Ihre Einblicke, Sie dürfen gern Ihre Erfahrungen und Gedanken teilen oder Bezug auf das nehmen, was in der Gruppe gesagt wird<br>- Wir wollen versuchen, ob es ohne tatsächliches oder „virtuelles“ Melden klappt. Sprechen Sie einfach frei los.	5 Min.

Appendix 3: Interview and Focus Group guides for Substudy 2

Focus Group guide for participants with expertise in clinical structures

Aufklärung	0	- Tonbandaufnahme + Aufnahme über Zoom (separat Video und Audio, Video wird direkt gelöscht)<br>- Wir werden in unseren Berichten keine Namen nennen und das, was Sie sagen, vertraulich behandeln.<br>- Die Tonaufnahme wird transkribiert und pseudonymisiert ausgewertet.<br>- Die Daten werden später verwendet, um das Konzept für die integrierte Versorgung auszuarbeiten<br>- Infos in Einwilligungserklärungen, dazu Fragen?<br>**ZETTEL & STIFT FÜR SPRECHERREIHENFOLGE BEREITHALTEN**	5
Vorstellung	0	Gut, fangen wir an.<br>Die Tonaufnahme startet jetzt. **TONBAND EINSCHALTEN**<br><br>- Vorstellung der Reihe nach<br>- Name<br>- Funktion<br>- Klinik<br>- Fachabteilung	5-10
Eröffnungsfrage: Erfahrungen mit psychosozialer Versorgung	1	**Die erste Frage bezieht sich auf Ihre Erfahrung mit bereits in Ihrer Klinik verfügbarer psychosozialer Versorgung. Was ist aus Ihren beruflichen Rollen heraus Ihre Erfahrung mit der Umsetzung psychosozialer Versorgung in Ihrer Klinik/Abteilung?**<br><br>Was ist Ihre Einstellung zu psychosozialen Versorgungskonzepten?<br><br>Einige von Ihnen haben bereits Erfahrungen mit dem Konzept der ins Team integrierten Stationspsychologin gemacht. Welche Erfahrungen haben Sie gemacht und wie haben Sie diese empfunden?<br><br>Was sind Ihre Erfahrungen mit den nun genannten Versorgungsformen? Wie werden diese angenommen, bzw. von den Mitarbeitenden als hilfreich erlebt?<br>- Was hat (/nicht) funktioniert?<br>- Was sind Vor-/Nachteile für die einzelnen Maßnahmen?<br>- Wie hat sich der Erfolg der Maßnahmen gezeigt?<br>- Was waren bisher auch Hürden/Hindernisse in der Etablierung der Maßnahme?<br>- Wie ließ sich die Versorgung finanzieren oder strukturell integrieren?	20

Appendix 3: Interview and Focus Group guides for Substudy 2

Focus Group guide for participants with expertise in clinical structures

Hauptfrage: Einschätzung der geplanten Intervention	2	**Das Ziel des IPS-Pilot ist die Entwicklung einer komplexen psychosozialen Intervention, welche unter anderem die Installation einer fest ins Team integrierten Stationspsychologin enthalten wird. Bisher ist geplant, eine Psycholog*in (M.Sc.) in Teilzeit, also 20 Wochenstunden, einzustellen, die sowohl für Mitarbeitende, Patient*innen und Angehörige niedrigschwellig verfügbar ist. Das heißt, sie ist direkt vor Ort auf der Station anwesend.**<br><br>**Wie ist Ihre Einschätzung aus Ihren beruflichen Perspektiven zu der vorgeschlagenen Intervention?**<br><br>Welche Chancen sehen Sie [aus Ihren beruflichen Perspektiven] für die vorgeschlagene Intervention?/ Welche Effekte der Intervention wären [aus Ihren beruflichen Perspektiven] wünschenswert?<br><br>Wie müsste die Intervention weiterhin aussehen, damit sie von Ihnen /[aus Ihren beruflichen Positionen] befürwortet wird?<br>Was an der Intervention würde hingegen eher abgelehnt werden oder gegen eine Etablierung sprechen?<br><br>Welche weiteren Rahmenbedingungen wären für die Umsetzung der Intervention notwendig/hilfreich? / Benötigt es organisatorische Voraussetzungen (z.B. Supervision) oder den Einbezug anderer Personen (Krankenkasse, Klinikvorstand, …)?<br><br>Ist die vorgeschlagene Intervention aus Ihren beruflichen Perspektiven machbar?<br>- Wenn ja, warum?<br>- Wenn nein, warum nicht?<br><br>○ Was denken die anderen?<br>○ Könnten Sie das näher ausführen? / Könnten Sie sagen, was genau Sie mit XY meinen?<br>○ Hätten Sie für XY ein Beispiel?	30 min
Hauptfrage 2: Weitere Maßnahmen		**Wären Ihrer Meinung nach neben einer Stationspsychologin noch weitere Maßnahmen notwendig, um die psychosoziale Versorgung zu gewährleisten?/In welchen Fällen bräuchte es noch weitere Maßnahmen?**<br><br>○ Was denken die anderen?	10

Appendix 3: Interview and Focus Group guides for Substudy 2

Focus Group guide for participants with expertise in clinical structures

		○ Könnten Sie das näher ausführen?/Könnten Sie sagen, was genau Sie mit XY meinen?<br>○ Hätten Sie für XY ein Beispiel?	
Ende	5	**Wir nähern uns dem Ende. Kommt noch etwas hinzu, das noch berücksichtigt werden sollte?**<br><br>**Okay, kommen wir zum Abschluss: Von all den Dingen, die wir besprochen haben, was ist für Sie das Wichtigste?**<br><br>**Möchte noch jemand ein abschließendes Wort sagen?**<br><br>- Bedankung<br>- Nochmal zur Erinnerung: Wenn Sie Interesse am Verlauf der Studie haben, können Sie eine E-Mail an ips.pilot@uniklinik-ulm.de schreiben<br><br>Tonaufnahme stoppen	5 Min.

,
