# Supplementary material for: Integrated Psychosocial Care in Intensive Care (IPS-Pilot): Protocol for the Systematic, Multimethod Development of a Complex Intervention (Phase A)
Source: JMIR Res Protoc. 2025 Jun 6;14:e65682. doi: 10.2196/65682 (PMC12181753; doi:10.2196/65682)
Supplement: Multimedia Appendix 4 [file resprot_v14i1e65682_app4.pdf]

Information für Probandinnen und Probanden

Titel der Studie: IPS-Pilot- Integrierte psychosoziale Versorgung in der Intensivmedizin

Sehr geehrte Probandin, sehr geehrter Proband,

wir möchten Sie bitten, an der folgenden Studie teilzunehmen: Das IPS-Pilot-Projekt zielt auf die Verbesserung der psychosozialen Situation in der Intensivmedizin für Mitarbeitende, Patient*innen und deren Angehörige ab. In der vorliegenden Studie führen wir Einzel- und Gruppeninterviews durch. Die Studie wird durch öffentliche Mittel (Innovationsausschuss des Gemeinsamen Bundesausschusses, Förderkennzeichen: 01VSF22020) gefördert. Die Studie wird an drei Standorten durchgeführt: an der Universitätsmedizin Magdeburg, an der Klinik für Psychosomatische Medizin und Psychotherapie des Uniklinikums Ulm sowie an der Medizinischen Klinik mit Schwerpunkt Psychosomatik der Charité Universitätsmedizin Berlin. Die Studie wurde von einer unabhängigen Ethikkommission geprüft.

Ausgangspunkt dieser Studie ist die Beobachtung, dass viele (ehemalige) Patient*innen ihren Intensivmedizinischen Aufenthalt als belastend erleben. Es wird zum Beispiel von Angst, Hoffnungslosigkeit, Albträumen oder Ähnlichem berichtet. Auch nach der Zeit auf der Intensivstation haben viele ehemalige Patient*innen noch Symptome von Muskelschwäche, Konzentrationsschwierigkeiten, Gedächtnislücken oder andere psychische Symptome. Auch für Angehörige kann diese Zeit stark belastend sein und zu psychischen Symptomen führen. Einige erleben aber auch sehr unterstützende Dinge, zum Beispiel große Hilfsbereitschaft, Unterstützung beim Füllen von Gedächtnislücken, haltgebende Bilder oder anderes.

Unser Ziel ist es, die Situation auf Intensivstationen für Patienten, deren Angehörigen sowie das Behandlungsteam zu verbessern. Dafür möchten wir psychologische Unterstützungsmöglichkeiten entwickeln, umsetzen und überprüfen. Von Ihnen wünschen wir uns Ihre Expertise als Betroffene*r Wir möchten gerne erfahren, welche Belastung oder auch welche Unterstützungen Sie während ihrer Zeit auf einer Intensivstation erlebt haben, und welche weiteren Ideen für Unterstützungsmöglichkeiten Sie haben. Nach Ihren Rückmeldungen zu den Bedarfen werden wir in weiteren Projektschritten in der Lage sein, eine psychosoziale Intervention zu entwickeln und mögliche Veränderungen zu implementieren, die dann zukünftigen Patient*innen, Angehörigen und Behandler*innen zu Gute kommen.

Der Studienablauf:

Bei unserem Termin werden wir - per Videokonferenz oder persönlich - ein Interview oder eine Fokusgruppe mit Ihnen führen, in dem es um Ihre Erlebnisse/Empfindungen und Beschwerden während Ihrer Arbeit auf der Intensivstation gehen soll. Das Interview wird ca. 30-60 Minuten dauern, eine Fokusgruppe ca. 60-90 Minuten. Das Gespräch wird aufgezeichnet. Aus der Teilnahme entstehen Ihnen keine Risiken. Sollte das Gespräch Sie aufgewühlt haben, können Sie sich bei Bedarf im Anschluss bei den Studienleitenden melden. Sollten Sie an den Ergebnissen der Interviews Interesse haben, senden wir Ihnen auf eine Aufforderung per E-Mail *ips.pilot@uniklinik-ulm.de* gern eine Zusammenfassung der analysierten Daten zu.

Freiwilligkeit

Ihre Teilnahme an diesem Forschungsprojekt ist freiwillig. Einwilligung können Sie jederzeit unter Angabe Ihres Pseudonyms und ohne Angabe von Gründen widerrufen. Alle bis dahin studienbedingt erhobenen Daten werden dann gelöscht.

Erreichbarkeit der Studienmitarbeiter

Sollten während des Verlaufs des Forschungsprojektes Fragen auftauchen, so können Sie diese jederzeit an folgenden Ansprechpartner richten:

Christian Hirning
E-Mail: *ips.pilot@uniklinik-ulm.de*

Versicherung

Während der Teilnahme an dem Forschungsprojekt genießen Sie Versicherungsschutz. Einen Schaden, der Ihrer Meinung nach auf dieses Forschungsprojekt zurückzuführen ist, melden Sie bitte unverzüglich ihrem lokalen Studienmitarbeiter.

Datenschutz

Rechtsgrundlage für die Verarbeitung Ihrer Daten ist Ihre freiwillige Einwilligung (Art. 6 Abs.1a, Art. 9 Abs. 2a DSGVO). Verantwortlich für die Datenverarbeitung sind die Klinik für Psychosomatische Medizin und Psychotherapie des Uniklinikums Ulm (Studienleiter: Prof. Harald Gündel), die Universitätsklinik für Psychosomatische Medizin und Psychotherapie der Otto-von-Guericke-Universität Magdeburg (Studienleiter: Prof. Florian Junne) , das Institut für Sozialmedizin und Gesundheitssystemforschung (Studienleiter: Prof. Apfelbacher) sowie die Medizinischen Klinik mit Schwerpunkt Psychosomatik der Charité Berlin (Studienleiter: Prof. Matthias Rose).

Datenverarbeitung

Alle im Rahmen der Studie erhobenen Daten über einzelne Personen werden streng vertraulich behandelt und nicht an Dritte außerhalb des Forschungsprojektes weitergegeben oder veröffentlicht. Alle Daten werden auf Computern gespeichert, die mit dem Internet verbunden sind. Ein legaler Zugriff auf diese Rechner von außerhalb ist nicht möglich.

Unsere erhobenen Daten beinhalten auch personenbezogene Daten wie ihr Geschlecht, Alter, Bildungsabschluss, etc. Nur die Klinik, an der Ihr Interview durchgeführt wird, hat Zugriff auf Ihre personenidentifizierenden Daten, ebenso wie die Vertrauensstelle, die zur Verwaltung dieser Daten beauftragt ist und ebenfalls strengen Datenschutzrichtlinien unterliegt. Bevor die gemeinsame Auswertung erfolgt, werden Ihre Daten pseudonymisiert, d.h. alle personenidentifizierenden Angaben, z.B. aus der Einwilligungserklärung, werden so verändert, dass ein Rückschluss auf Sie als Person bestmöglich verhindert wird. Pseudonymisieren bedeutet, dass die personenbezogenen Daten wie das Alter ohne Hinzuziehung einer Liste nicht mehr einer konkreten Person zugeordnet werden können. Die personenbezogenen Daten werden durch einen Nummern- und Buchstabencode ersetzt. Die pseudonymisierten Daten werden zum Zweck der wissenschaftlichen Auswertung weitergeleitet.

Im Übrigen werden alle personenbezogenen und pseudonymisierten Daten durch die Vertrauensstelle an der Biometrie der Otto-von-Guericke Universität Magdeburg (E-Mail: *ths@med.ovgu.de*) verwaltet und nach Ablauf von 10 Jahren gelöscht.

Das Interview wird mit einem Aufnahmegerät aufgezeichnet. Die Tonaufnahme des Interviews wird zunächst an der durchführenden Klinik gespeichert. Das Gespräch wird innerhalb von drei Monaten wortwörtlich aufgeschrieben (transkribiert). Dies geschieht durch das Schreibbüro amanu GmbH. Bei der Transkription wird jeder Hinweis auf Ihre Identität unkenntlich gemacht. Die Tonaufnahmen werden nach Ablauf von 10 Jahren gelöscht.

Schweigepflicht

Alle Personen, die an der Durchführung dieser Interviews beteiligt sind, unterliegen der beruflichen Schweigepflicht und sind auf das Datengeheimnis verpflichtet. Die studienbezogenen Untersuchungsergebnisse sollen in anonymisierter Form in wissenschaftlichen Veröffentlichungen verwendet werden. Soweit es zur Kontrolle der korrekten Datenerhebung erforderlich ist, dürfen autorisierte Personen (z.B. des Auftraggebers, der Universität) Einsicht in die studienrelevanten Teile der Akte nehmen. Sofern zur Einsichtnahme autorisierte Personen nicht der obengenannten beruflichen Schweigepflicht unterliegen, stellen personenbezogene Daten, von denen sie bei der Kontrolle Kenntnis erlangen, Betriebsgeheimnisse dar, die geheim zu halten sind.

Welche weiteren Rechte habe ich bezogen auf den Datenschutz?

Sie haben das Recht, Auskunft über die Sie betreffenden Daten zu erhalten, auch in Form einer unentgeltlichen Kopie.

Darüber hinaus können Sie die Berichtigung Ihrer Daten verlangen oder die Teilnahme widerrufen. Wenden Sie sich in diesen Fällen bitte an die unabhängige Vertrauensstelle:

E-Mail: *ths@med.ovgu.de*

<u>Vertrauensstelle</u>
Otto-von-Guericke Universität Magdeburg
Medizinische Fakultät
Leipziger Str.44
39120 Magdeburg

Tel.: +49 391 67-24362

Für inhaltliche Fragen zu Studienergebnissen wenden Sie sich bitte an die zentrale Studienleitung: *ips.pilot@uniklinik-ulm.de*

Bei Rückfragen zum Datenschutz wenden Sie sich bitte an die Datenschutzbeauftragten. Falls Sie Bedenken oder Beschwerden hinsichtlich der Verarbeitung Ihrer Daten haben, wenden Sie sich bitte an die Datenschutz-Aufsichtsbehörde Ihres Studienzentrums (siehe Kontaktdaten Tabelle).

<table>
<tr><th colspan="2">Datenschutzbeauftragte</th><th>Datenschutz-Aufsichtsbehörde</th></tr>
<tr><td>Berlin</td><td>Datenschutzbeauftragte der Charité – Universitätsmedizin Berlin
Charitéplatz 1
10117 Berlin
Telefon: +49 30 450 580016
E-Mail: datenschutzbeauftragte@charite.de</td><td>Berliner Beauftragte für Datenschutz und Informationsfreiheit
Alt-Moabit 59-61
10555 Berlin
Telefon: +49 30 13889-0
Fax: +49 30 2155050
E-Mail: mailbox@datenschutz-berlin.de</td></tr>
<tr><td>Magdeburg</td><td>Datenschutzbeauftragte der Universitätsmedizin Magdeburg (Medizinische Fakultät der Otto-von-Guericke-Universität Magdeburg und Universitätsklinikum Magdeburg A.ö.R.)

Universitätsklinikum Magdeburg A.ö.R.
Leipziger Str. 44
39120 Magdeburg

+49-391-67-15753

datenschutz@med.ovgu.de</td><td>Der Datenschutzbeauftragte des Landes Sachsen-Anhalt

Geschäftsstelle/Besucheradresse:
Leiterstr. 9; 39104 Magdeburg
Postadresse:
Postfach:1947, 39009 Magdeburg

+49-391-81803-0
+49-391-81803-33

poststelle@fd.sachsen-anhalt.de</td></tr>
<tr><td>Ulm</td><td>Datenschutzbeauftragter des Universitätsklinikums Ulm:
Albert-Einstein-Allee 29
89081

0731 / 500-69290, E-

dsb.ukl@uniklinik-ulm.de</td><td>Der Landesbeauftragte für den Datenschutz und die Informationsfreiheit Baden-Württemberg

Lautenschlagerstraße 20
70173 Stuttgart

www.baden-wuerttemberg.datenschutz.de/kontakt-aufnehmen/</td></tr>
</table>

Einwilligungserklärung

Titel der Studie: IPS-Pilot- integrierte psychosoziale Versorgung in der Intensivmedizin

Inhalt, Vorgehensweise, Risiken und Ziel des oben genannten Forschungsprojektes sowie die Befugnis zur Einsichtnahme in die erhobenen Daten hat mir ________________________ ausreichend erklärt.

Ich hatte zusätzliche Fragen:

____

Ich hatte Gelegenheit Fragen zu stellen und habe hierauf Antwort erhalten.

Ich hatte ausreichend Zeit, mich für oder gegen die Teilnahme am Projekt zu entscheiden.

Eine Kopie der Probandeninformation und Einwilligungserklärung habe ich erhalten.

Mit der Aufzeichnung auf Audiotape bin ich einverstanden.

Ich willige in die Teilnahme am Forschungsprojekt ein:

____
(Name Proband*in)

____________________________ ____________________________
Ort, Datum (Unterschrift Proband*in)

Erklärung und Unterschrift der aufklärenden Ärzt*in / Psycholog*in:
Ich habe das Aufklärungsgespräch geführt und die Einwilligung eingeholt.

____
(Name und Vorname in Druckschrift)

_______________________________ ________________________
(Datum) (Unterschrift)

Information und Einwilligungserklärung zum Datenschutz

Bei wissenschaftlichen Studien werden persönliche Daten und medizinische Befunde über Sie erhoben. Die Speicherung, Auswertung und Weitergabe dieser studienbezogenen Daten erfolgt nach gesetzlichen Bestimmungen und setzt vor Teilnahme an der Studie folgende freiwillige Einwilligung voraus:

1. Ich erkläre mich damit einverstanden, dass im Rahmen dieser Studie erhobene Daten/ Krankheitsdaten auf Fragebögen und elektronischen Datenträgern aufgezeichnet und ohne Namensnennung verarbeitet werden.
2. Außerdem erkläre ich mich damit einverstanden, dass eine autorisierte und zur Verschwiegenheit verpflichtete Person (z.B.: des Auftraggebers, der Universität) in meine erhobenen personenbezogenen Daten Einsicht nimmt, soweit dies für die Überprüfung des Projektes notwendig ist. Für diese Maßnahme entbinde ich die Ärztin / den Arzt von der beruflichen Schweigepflicht.
3. Ich habe verstanden, dass ich das Recht habe, Auskunft (einschließlich unentgeltlicher Überlassung einer Kopie) über die mich betreffenden personenbezogenen Daten zu erhalten sowie deren Berichtigung oder Löschung zu verlangen.

Ich willige in die oben beschriebene Verwendung meiner Daten ein:

____

(Name Proband*in)

______________________________ ______________________________

Ort, Datum (Unterschrift Proband*in)

Pseudonym: ___________________
